# DREAM: an R package for druggability evaluation of human complex diseases

**DOI:** 10.1093/bioinformatics/btad442

**Published:** 2023-07-20

**Authors:** Antonio Federico, Michele Fratello, Alisa Pavel, Lena Möbus, Giusy del Giudice, Angela Serra, Dario Greco

**Affiliations:** Finnish Hub for Development and Validation of Integrated Approaches (FHAIVE), Faculty of Medicine and Health Technology, Tampere University, Tampere 33100, Finland; Tampere Institute for Advanced Study, Tampere University, Tampere 33100, Finland; Division of Pharmaceutical Biosciences, Faculty of Pharmacy , University of Helsinki, Helsinki 00100, Finland; Finnish Hub for Development and Validation of Integrated Approaches (FHAIVE), Faculty of Medicine and Health Technology, Tampere University, Tampere 33100, Finland; Finnish Hub for Development and Validation of Integrated Approaches (FHAIVE), Faculty of Medicine and Health Technology, Tampere University, Tampere 33100, Finland; Finnish Hub for Development and Validation of Integrated Approaches (FHAIVE), Faculty of Medicine and Health Technology, Tampere University, Tampere 33100, Finland; Finnish Hub for Development and Validation of Integrated Approaches (FHAIVE), Faculty of Medicine and Health Technology, Tampere University, Tampere 33100, Finland; Division of Pharmaceutical Biosciences, Faculty of Pharmacy , University of Helsinki, Helsinki 00100, Finland; Finnish Hub for Development and Validation of Integrated Approaches (FHAIVE), Faculty of Medicine and Health Technology, Tampere University, Tampere 33100, Finland; Tampere Institute for Advanced Study, Tampere University, Tampere 33100, Finland; Division of Pharmaceutical Biosciences, Faculty of Pharmacy , University of Helsinki, Helsinki 00100, Finland; Finnish Hub for Development and Validation of Integrated Approaches (FHAIVE), Faculty of Medicine and Health Technology, Tampere University, Tampere 33100, Finland; Tampere Institute for Advanced Study, Tampere University, Tampere 33100, Finland; Division of Pharmaceutical Biosciences, Faculty of Pharmacy , University of Helsinki, Helsinki 00100, Finland; Institute of Biotechnology, University of Helsinki, Helsinki 00100, Finland

## Abstract

**Motivation:**

*De novo* drug development is a long and expensive process that poses significant challenges from the design to the preclinical testing, making the introduction into the market slow and difficult. This limitation paved the way to the development of drug repurposing, which consists in the re-usage of already approved drugs, developed for other therapeutic indications. Although several efforts have been carried out in the last decade in order to achieve clinically relevant drug repurposing predictions, the amount of repurposed drugs that have been employed in actual pharmacological therapies is still limited. On one hand, mechanistic approaches, including profile-based and network-based methods, exploit the wealth of data about drug sensitivity and perturbational profiles as well as disease transcriptomics profiles. On the other hand, chemocentric approaches, including structure-based methods, take into consideration the intrinsic structural properties of the drugs and their molecular targets. The poor integration between mechanistic and chemocentric approaches is one of the main limiting factors behind the poor translatability of drug repurposing predictions into the clinics.

**Results:**

In this work, we introduce DREAM, an R package aimed to integrate mechanistic and chemocentric approaches in a unified computational workflow. DREAM is devoted to the druggability evaluation of pathological conditions of interest, leveraging robust drug repurposing predictions. In addition, the user can derive optimized sets of drugs putatively suitable for combination therapy. In order to show the functionalities of the DREAM package, we report a case study on atopic dermatitis.

**Availability and implementation:**

DREAM is freely available at https://github.com/fhaive/dream. The docker image of DREAM is available at: https://hub.docker.com/r/fhaive/dream.

## 1 Introduction

The design, synthesis, and preclinical testing of new drugs are long and expensive, significantly delaying their introduction to the market and, in turn, into clinical practice (Pammolli *et al.* 2011). This posed the need for re-usage of approved drugs, designed for other therapeutic indications. Such an approach, known as drug repurposing, became a fiery field of research in the last decade (Pushpakom *et al.* 2019). However, in order to achieve reliable drug repurposing predictions, a thorough druggability evaluation of the disease under study is necessary. To date, the druggability is assessed mainly by three different approaches that are poorly integrated. First, profile-based approaches are based on the wealth of data available in public repositories about drug sensitivity profiles, allowing the characterization of thousands of mechanisms of action (MOAs) of drugs at different treatment conditions. Large scale repositories such as LINCS1000 (Stathias *et al.* 2020), Open TG-Gates (Igarashi *et al.* 2015), and DrugMatrix (Ganter *et al.* 2006) allowed a deeper understanding of pharmacologic perturbations at a molecular scale. Second, network-based approaches focus their attention mainly on the impaired molecular interactions underlying a certain disease and, given their properties, they represent an unprecedented opportunity to fill the knowledge gaps about the disease under consideration. Third, structure-based approaches point their attention to the intrinsic structural properties of drugs and their molecular targets, uncovering the binding mechanisms at atomic level between macromolecules and exogenous compounds, and allowing the *de novo* prediction of drug–target interactions. However, taken as standalone entities, these approaches present some limitations. For instance, profile-based approaches neglect the intrinsic properties of the drugs, and the molecular make-up underlying the disease under investigation. Network-based approaches alone do not take into consideration the MOA of drugs and their structural properties. Structure based approaches, instead, are limited because they do not account for the wide unbalance underlying human diseases and the complex range of molecular perturbations triggered by a drug treatment.

Here, we introduce DREAM, an R package that integrates mechanistic and chemocentric approaches in a unified computational workflow. DREAM is devoted to druggability evaluation of pathological conditions of interest, leveraging robust drug repurposing, and drug combination predictions. Our framework provides several functionalities, such as (i) identification of disease-relevant genes and inference of co-expression networks, (ii) identification of candidate drug targets, (iii) inference and evaluation of drugs MOA, (iv) evaluation of drug–drug similarities based on their MOA and/or chemical structure, and (v) identification of drug candidates to repurposing and combination therapy for the pharmacological treatment of the disease under consideration. DREAM is suitable for evaluating the druggability and possible drug combinations for any human disease. Here, we showcase the functionalities of DREAM by carrying out a case study on atopic dermatitis, a chronic inflammatory skin condition characterized by dry, itchy, and inflamed skin. Its pathogenesis results from a combination of genetic and environmental factors, including impaired skin barrier function, immune system dysregulation, and an increased tendency towards allergic reactions (Möbus *et al.* 2021). While current treatment options, such as topical corticosteroids and immunomodulators, are effective for many patients, developing novel pharmacological treatments for atopic dermatitis is crucial for improving symptom control, overcoming treatment resistance, minimizing side effects, and targeting disease mechanisms (Adikusuma *et al.* 2021). These advancements can significantly enhance the management of atopic dermatitis and improve patients’ lives.

## 2 Implementation

**Figure 1.**
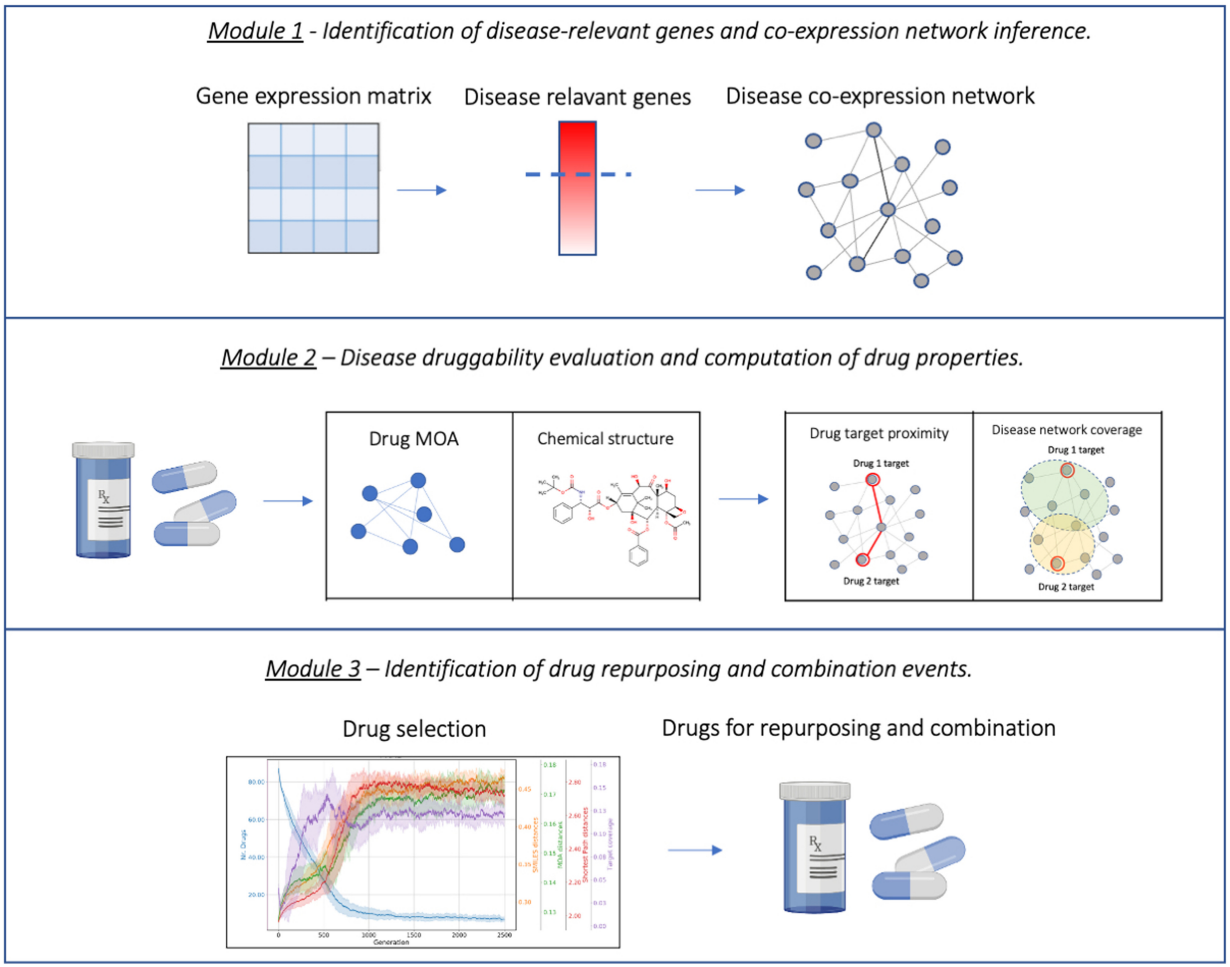
Overview of the functionalities of the DREAM package. In Module 1, the user can identify disease-relevant genes and infer a co-expression network or utilize a ready-made interactome. In Module 2, the user can evaluate the druggability of the disease under study and calculate several properties of the drugs and topological properties of their targets. In Module 3, the user can derive drug repurposing predictions and drug combinations based on the properties calculated in the previous module.

The DREAM package consists of three modules (Fig. 1, Supplementary File S1). In Module 1, the user inputs gene expression data (derived from DNA microarray or RNA-Sequencing technologies) and retrieves genes that are relevant to the disease under consideration. To extrapolate such genes, DREAM implements a Wilcoxon test to assess substantial gene expression differences between the disease and the healthy counterpart and an F-test on the variance to test the gene expression stability between the two conditions. Moreover, in Module 1 the user can build a gene co-expression network underlying the disease or, alternatively, can use a pre-built co-expression network (or any other interactome, such as a protein–protein interaction) to perform operations implemented in the next modules. DREAM implements functions for the inference and analysis of robust biological networks. The network can be inferred by combining multiple network inferences from an ensemble of methods [as reported in Marwah *et al.* (2018)]. In Module 2, the user can evaluate the druggability of the disease under study. Several functions are implemented in order to assess drug–drug similaritiy based on several criteria, including MOA, chemical (sub-)structures, and topological characteristics of drug targets on the disease network. In detail, DREAM enables the comparison of drugs based on their MOA, by calculating a pairwise Hamming–Ipsen–Mikhailov distance between the drug perturbational networks. Drugs can be, then, compared by their chemo-structural properties. The users can compute ECFP fingerprints for the drugs of interest and compare their chemical substructures by calculating a Tanimoto similarity or their Maximal Common Substructure. Moreover, DREAM can extrapolate drug structures in the form of SMILES (Simplified Molecular Input Line Entry System) and calculate a pairwise Levenstein distance. In Module 2, the user can also evaluate topological properties of the drugs on the disease network, such as the area of action of the drugs under investigation on the disease network and the distance between their drug targets. The area of action for a drug is defined as the union of target genes and their direct interactors normalized on the total number of nodes composing the disease network, while the distances between drug targets of pairs of drugs are expressed in terms of shortest paths. For drugs having more than one target in the disease network, then the average shortest path of all the possible target pairs of the two drugs is computed. Based on such evaluations, the user can investigate drug–drug synergies for the biological system under study. In Module 3, the user can exploit the results obtained in the previous modules in order to perform a drug selection aimed to identify drugs suitable for repurposing and combination. The selection is performed by means of a multi-objective genetic algorithm (GA). The GA in the DREAM package is set to optimize five objective functions: number of drugs in the combination, MOA, secondary structure, coverage of the disease network, and drug–target distance. A general overview of the functionalities implemented in each module of the DREAM package is shown in Fig. 1.

## 3 Application

In order to showcase the functionalities of DREAM, we integrate profile-based, network-based, and structure-based methods to evaluate the druggability, the potential drug repurposing, and the candidate therapeutic combinations for atopic dermatitis (Supplementary File S2). In our case study, we exploited preprocessed and harmonized transcriptomics datasets [data stored in Zenodo, DOI: 10.5281/zenodo.4009497 (Federico *et al.* 2020)] of both lesional and unaffected skin of atopic dermatitis patients. First, we identified genes whose expression is altered in the atopic lesion with respect to the unaffected skin. Such genes were utilized to build a co-expression network representing the atopic lesion. Second, we characterized the MOA of 21 drugs tested in FIBRNPC cell line by generating co-expression networks of perturbational profiles collected from the LINCS1000 repository (Subramanian *et al.* 2017). Third, we extrapolated the chemical structure/substructures of the considered drugs by collecting their structural representations by PubChem (https://pubchem.ncbi.nlm.nih.gov/). Therefore, by integrating these three layers of information through the functionalities of DREAM, we modelled the druggability of the atopic dermatitis lesion. In addition to drugs that are already in use for the treatment of noncancerous skin diseases and that are included in clinical trials, such as caffeine, calcitriol, and calciferol (Kaplan *et al.* 1977, 1978, Siegel *et al.* 1978, Berents *et al.* 2016, Weinhold *et al.* 2016, Bothou *et al.* 2018, Sánchez-Armendáriz *et al.* 2018, Alashqar 2019, Tukaj *et al.* 2019), we identified suramin, risperidone, and metformin as potential candidates for repurposing since these drugs have never been utilized nor tested in the treatment of atopic lesions. Moreover, by investigating the structural, functional, and topological characteristics of the drugs included in the study, we derived drug combinations that could be further investigated to improve the therapeutic outcome for atopic dermatitis. Our drug combination prediction led to the identification of 20 combinations composed of 2 drugs, 17 combinations composed of 3 drugs, and 4 combinations composed of 4 drugs for atopic dermatitis.

## 4 Discussion

Despite multiple computational strategies have been developed in the realm of predictive pharmacology to improve therapeutic approaches for complex diseases, the predictions rarely reached the clinical application.

One of the reasons behind this drawback is that current predictive approaches are based only on the integration of molecular perturbation of a certain disease with drug sensitivity signatures, neglecting intrinsic properties of the drugs. To date, several algorithms for druggability evaluation, drug repurposing, and combination prediction have been developed (Gottlieb *et al.* 2011, Huang *et al.* 2014, Wang *et al.* 2022). On one hand, most of these algorithms rely only on the molecular dysregulation underlying the disease under investigation or the structural characteristics of the drugs, capturing only a limited aspect of the druggability process (Supplementary File S2). For instance, the intrinsic mechanism of action of the drugs along with the interplay between their drug targets, is often neglected. On the other hand, many of the existing tools do not make the code available to the users, often allowing only noncustomizable analyses. We therefore developed DREAM, which includes several exposed functions applicable for different purposes spanning from simple druggability evaluations to more advanced drug combination predictions. The information generated by DREAM can help to find therapeutic alternatives for complex diseases, evaluating the chemical similarity, shared therapeutic targets, drug mechanism of action, or any of their combinations, according to the pharmacological context. This allows the users to customize their analytical pipeline based on the specific problem to be solved.

## 5 Conclusions

We developed DREAM, an R package that integrates mechanistic and chemocentric approaches to drug repurposing and drug combination predictions. Network-based, profile-based, and structure-based approaches are integrated in a unique framework, allowing the user to evaluate several aspects of the druggability of the disease of interest. The applicability, together with all of the functionalities of DREAM were highlighted in the performed case study.

## Supplementary Material

btad442_Supplementary_Data
