# Supplementary material for: DREAM: an R package for druggability evaluation of human complex diseases
Source: Bioinformatics. 2023 Jul 20;39(7):btad442. doi: 10.1093/bioinformatics/btad442 (PMC10374489; doi:10.1093/bioinformatics/btad442)
Supplement: btad442_Supplementary_Data [file btad442_supplementary_data.zip › Supplementary_file_1(1).docx]

## DREAM: an R package for druggability evaluation of human complex diseases.

Antonio Federico^1,2,†^, Michele Fratello^1,†^, Alisa Pavel^1^, Lena Möbus^1^, Giusy del Giudice^1^, Angela Serra^1,2^, Dario Greco^1,2,3,4,*^

^1^ Finnish Hub for Development and Validation of Integrated Approaches (FHAIVE), Faculty of Medicine and Health Technology, Tampere University, 33100 Tampere, Finland

^2^ Tampere Institute for Advanced Study

^3^ Division of Pharmaceutical Biosciences, Faculty of Pharmacy, University of Helsinki, 00100 Helsinki, Finland

^4^ Institute of Biotechnology, University of Helsinki, 00100 Helsinki, Finland

^*^ Author to whom correspondence should be addressed.

^†^ These authors contributed equally to this work.

Implementation

DREAM is an R package organised in 3 modules (Figure S1). This package is suitable for researchers looking for an easy solution to evaluate druggability predictions for complex diseases for which gene expression deriving from transcriptomics experiments is available. Following are reported Bioconductor, CRAN R and python packages used in DREAM:

- stats >= 3.6.0 [(R Core Team 2019)](https://sciwheel.com/work/citation?ids=14503047&pre=&suf=&sa=0)
- igraph >= 1.2.4.1 [(Csardi & Nepusz 2006)](https://sciwheel.com/work/citation?ids=13648543&pre=&suf=&sa=0)
- S4Vectors >= 0.22.0 [(H. Pagès, M. Lawrence and P. Aboyoun 2019)](https://sciwheel.com/work/citation?ids=14498469&pre=&suf=&sa=0)
- utils >= 3.6.0 [(R Core Team 2019)](https://sciwheel.com/work/citation?ids=14503047&pre=&suf=&sa=0)
- XML >= 3.98-1.2 [(Duncan Temple Lang and the CRAN Team 2019)](https://sciwheel.com/work/citation?ids=14498491&pre=&suf=&sa=0)
- Rcurl >= 1.95-4.1 [(Duncan Temple Lang and the CRAN team 2019)](https://sciwheel.com/work/citation?ids=14503069&pre=&suf=&sa=0)
- parallel >= 3.6.0 [(R Core Team 2019)](https://sciwheel.com/work/citation?ids=14503047&pre=&suf=&sa=0)
- doParallel >= 1.0.14 [(Microsoft Corporation and Steve Weston 2018)](https://sciwheel.com/work/citation?ids=14498519&pre=&suf=&sa=0)
- foreach >= 1.4.4 [(Microsoft and Steve Weston 2017)](https://sciwheel.com/work/citation?ids=14498528&pre=&suf=&sa=0)
- minet >= 3.42.0 [(Meyer et al. 2008)](https://sciwheel.com/work/citation?ids=950850&pre=&suf=&sa=0)

Python packages:

- python=3.7.12
- deap=1.3.1 [(De Rainville et al. 2012)](https://sciwheel.com/work/citation?ids=7565056&pre=&suf=&sa=0)
- rdkit=2022.03.3 [(Tosco et al. 2014)](https://sciwheel.com/work/citation?ids=14498608&pre=&suf=&sa=0)
- flask=2.1.2 [(Grinberg, M. 2018)](https://sciwheel.com/work/citation?ids=14498864&pre=&suf=&sa=0)
- requests=2.27.1 [https://github.com/kennethreitz/requests]
- python-igraph=0.9.11 [(Csardi & Nepusz 2006)](https://sciwheel.com/work/citation?ids=13648543&pre=&suf=&sa=0)
- pyreadr=0.4.7 [https://github.com/ofajardo/pyreadr]

Additional packages used for the analysis of the results

- matplotlib=3.5.3 [(Hunter 2007)](https://sciwheel.com/work/citation?ids=1310480&pre=&suf=&sa=0)
- seaborn=0.12.2 [(Waskom 2021)](https://sciwheel.com/work/citation?ids=11026692&pre=&suf=&sa=0)
- scikit-learn=1.0.2 [(Pedregosa et al., 2011)](https://sciwheel.com/work/citation?ids=14498897&pre=&suf=&sa=0)

***Module 1 - Identification of disease-relevant genes and co-expression network inference.***

In module 1, in order to identify genes that are significantly dysregulated in the disease with respect to the control samples, the user is requested to give in input a gene expression matrix deriving from a transcriptomics experiment (DNA microarray or bulk RNA-Seq), having genes on the rows and samples on the columns. The user should also indicate the biological conditions of each sample in order to identify disease-relevant genes. The function “*get_informative_genes*” allows the user to perform a non-parametric hypothesis test (Wilcoxon test) in order to identify substantial differences between the disease samples and the controls. On the other hand, the user can choose to perform a test on the expression variance across samples of the biological conditions. The criterion behind this test is that the phenotype underlying a pathological status, is not only reflected by differential expression of the genes, but also by disrupted regulatory mechanisms that affect the expression stability of certain genes in the disease population with respect to the healthy counterpart. Once the disease-relevant genes are identified, the user can build a co-expression network of such a robust set. Such a network, named by now on Disease Network (DN), will be the canvas on which the evaluations implemented in the next modules will be performed. Alternatively, the user can build a co-expression network with a set of genes of choice or utilise a pre-built co-expression network (Figure S1).


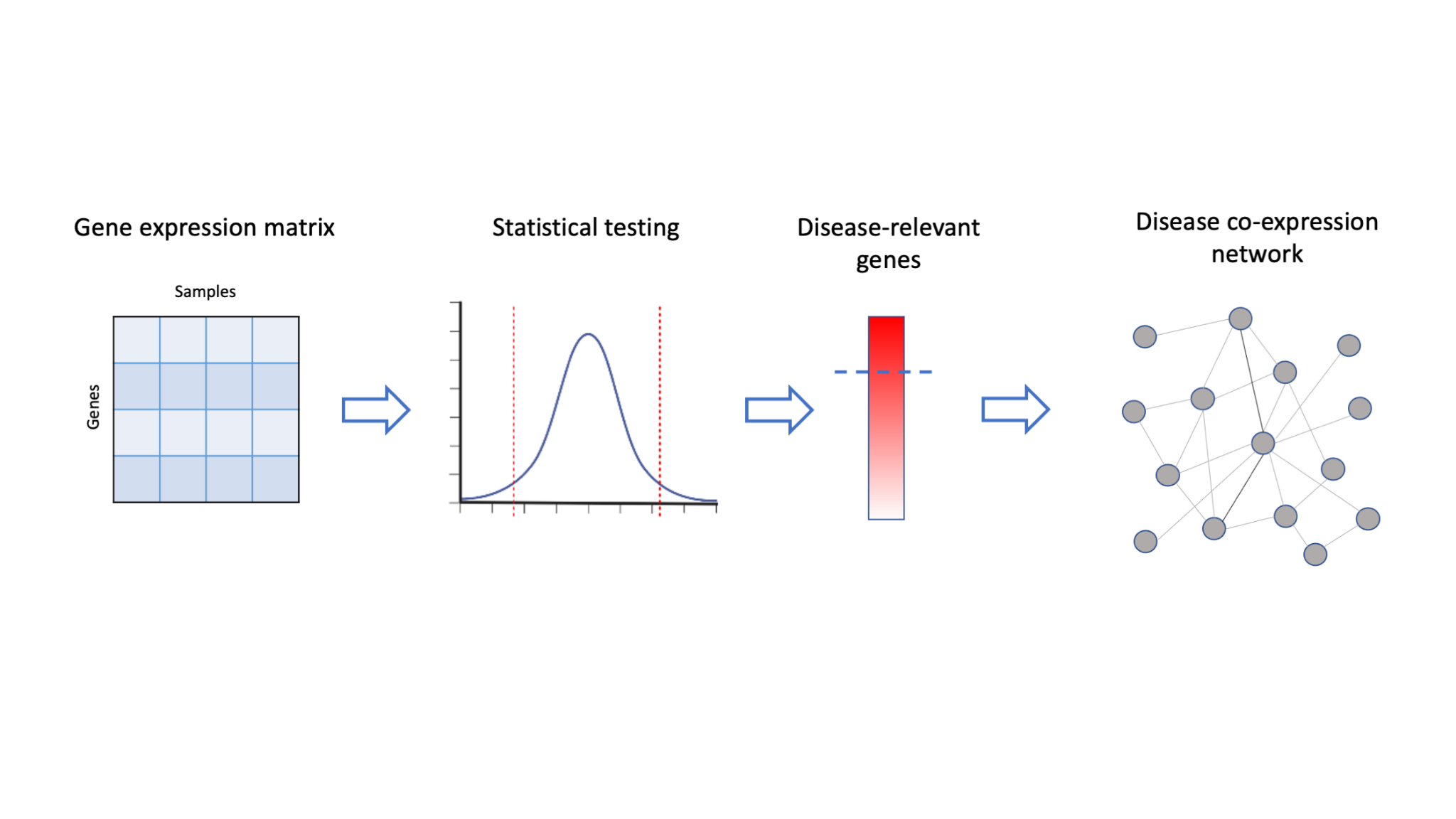


*Figure S1 - Framework implemented in Module 1 of the DREAM package. The user can perform a gene expression analysis to identify disease-relevant genes and subsequently build a co-expression network.*

***Module 2 – Druggability evaluation of the disease under investigation and computation of drug properties.***

In Module 2, the druggability of the biological system under investigation is evaluated. The first step consists in identifying drug-target associations on the DN. The drugs whose targets are nodes of the DN are identified based on the OpenTarget annotation (version 19.02). The subsequent evaluations implemented in Module 2 are focused on both intrinsic properties of the drugs and topological properties of the drug targets on the DN. In particular, while the DREAM package takes into consideration the MOA and the chemical (sub-)structures as drug properties, it also considers properties of the drug target in the biological system under consideration, such as the distance of drug targets in the DN and the area of action of the drug on the DN. The following paragraphs explain in detail the evaluations implemented in Module 2 (Figure S2).

*Module 2.1 - Characterisation and comparison of drug mechanism of action (MOA).*

Several publicly available databases are currently available that report large-scale drug sensitivity profiles on a considerable amount of cell lines and tissues. For instance, LINCS1000 contains about 1,328,098 gene expression profiles resulting from the application of 42,553 perturbagens (19,811 small molecule compounds, 18,493 shRNAs, 3,627 cDNAs, and 622 biologics) for a total of 476,251 signatures. Similarly, the Open TG-Gates is a repository dedicated to toxicological effects of a plethora of drugs, whose effect has been tested in rat liver. The wealth of data available in these repositories represents an unprecedented possibility to investigate both the therapeutic and toxicological effects of drug treatments in different experimental conditions, facilitating the prediction of drug repurposing events, drug adverse effects or drug synergy. In this module, we exploit this big amount of information in order to characterise the MOA of drugs and exploit its potential in order to compare drugs.

The MOA of drugs is defined as the molecular perturbation that a certain drug treatment induces on a biological system. In this study, the MOA of drugs is represented as co-expression networks inferred from the transcriptional perturbation induced by treatment of a certain cell line with several drugs. Therefore, in this module, we build a co-expression network for each of the drugs under investigation in order to define their MOA, and calculate a matrix of similarity among the drugs based on the Hamming-Ipsen-Mikhailov (HIM) distance [(Jurman et al. 2015)](https://sciwheel.com/work/citation?ids=7910061&pre=&suf=&sa=0).

*Module 2.2 - Characterisation and comparison of drug chemical substructures.*

Based on the general assumption that the structure makes the function, this module is aimed to characterise and compare the chemical structures and substructures of drugs that are mapped onto the network. In this framework, the user has the possibility of analysing chemical structure either directly in the form of SMILES strings retrieved by OpenTargets or molecular graphs. If SMILES are considered, then the drug-drug similarity is computed through the Levenstein distance. On the other hand, if molecular graphs are used the similarity between a pair of drugs can be evaluated in two different ways: I) fingerprint similarity: ECFP fingerprints for each drug are computed and then the Tanimoto similarity of the substructures is evaluated; II) Maximal Common Substructure (MCS): for each pair of drugs, their similarity is determined by the number of shared atoms (determined by computing the MCS of the two drugs) divided by the total number of atoms in the two drugs.


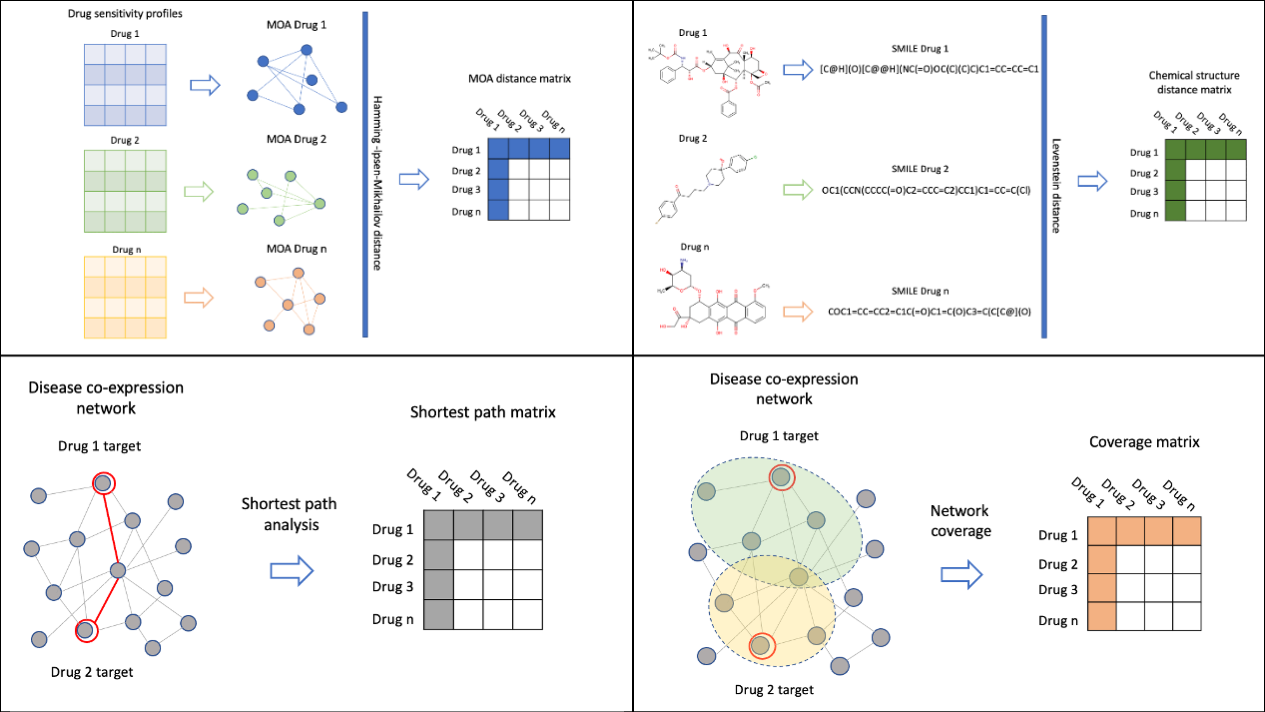


*Figure S2 - Functionalities embedded in Module 2 of the DREAM package. The user can choose to run one or more of the implemented functions, in order to evaluate the druggability of the disease of interest.*

*Module 2.3 - Evaluation of the DN coverage.*

In Module 2 the user can evaluate the area of action of the drugs under investigation on the DN. The area of action for a drug was defined as the group of genes including the direct interactors of each of the target genes. Eventually, a matrix reporting the coverage of pairs of drugs is computed. Specifically, the coverage is defined as the non-overlapping area of the DN in terms of direct interactors of drug targets normalised on the total number of nodes composing the DN.

*Module 2.4 - Evaluation of drug target distances on the disease network.*

The function “*get_avg_shortest_path”* allows the user to build a matrix of the distances between drug targets of pairs of drugs on the DN. If one (or both) drug(s) of the pair have more than one target in the DN, then the average of the distances of all the possible target pairs of the two drugs is computed. The distances of the drug targets on the DN are expressed in terms of shortest paths.

*Module 3 – Identification of drug combinations.*

In Module 3, the user can optionally identify sets of drugs that are suitable for repurposing and combination for the phenotype under study, based on the druggability evaluations performed in Module 2. The selection is performed by means of a multi-objective genetic algorithm (GA). GAs are well established tools to solve combinatorial optimization problems (Figure S3).

In multi-objective optimization there is not a single optimal solution but rather a set that cannot be further improved in any of the objective functions, without reducing the quality of at least one other objective. These solutions are called non-dominated solutions and the set of all non-dominated solutions is called the Pareto front.

The GA in the DREAM package is set to optimise 5 objective functions as follows:

Number of drugs in the combination: The first function is the number of drugs in the solutions. The GA is set to select the minimum number of drugs to combine in order to achieve the therapeutic effect, while significantly reducing the toxicity profile due to the treatment.

MOA: In order to achieve the maximum therapeutic effect by means of combination therapy, functional redundancy should be avoided among drugs included in the same solution. To satisfy this condition, the GA exploits the distance matrix computed on the drug MOAs (Module 2.1), by prioritising combinations of drugs showing the most different MOA.

Secondary structure: As stated before, the DREAM package is based on the concept that the chemical structure affects the function. In this regard, drugs having similar structure are likely to have similar mechanisms of action. As well, drugs that share functional groups, represented by chemical substructures, are prone to share functional similarities. Therefore, as for the case of the MOA, also for the chemical structure the GA is set to prioritise drugs with the most different chemical (sub-)structures.

Drug target distance: The GA takes into consideration the distance among drug targets on the DN. In order to maximise the therapeutic effect of the drug combination, the action of drugs involved in a combination therapy should ideally be exerted on non-overlapping areas of the DN. In this regard, the GA prioritises drugs showing the longest distance (in terms of shortest path) in the disease network among their targets.

Disease network coverage: Similarly, in order to achieve the maximum therapeutic effect with the minimum amount of drugs, the GA included in the DREAM package is set so as to prioritise drug combinations that maximise the coverage of the DN, calculated as described above (Module 2.4).

We model a unique solution of the problem, also called a chromosome, as a binary vector $x \in\{0, 1{\}}^{n}$, where $n$ is the total number of drugs considered in the problem. A value of 1 means that the drug is considered part of the solution, while 0 means that the corresponding drug does not contribute to the solution.

During initialization, a population $P$ of $k_{pop}$ individual solutions are randomly generated. Specifically, each bit in each solution has a tunable chance $p_{init}$ of being selected. After initialization, the following steps are repeated for a number of iterations specified by the user:

1. Generation of the offspring: a number of $k_{off}$ new individuals are generated by applying either the crossover operator, the mutation operator, or the cloning operator. The probabilities of which operator to apply are chosen by the user and must sum to 1. More in detail,
   - The crossover operator chooses two individuals at random from the population and creates a new individual by first selecting a point in the parents’ chromosomes and then swapping the values to the right side of the crossover point between the two parents. In our implementation, only the first child chromosome is kept, while the second is discarded.
   - The mutation operator randomly switches individual attributes within one individual chromosome. Each element of the solution has an independent probability $p_{mut}$ to be switched to another position.
   - The cloning operator randomly chooses one individual for the parent population and creates a clone of it into the offspring.
2. Evaluation of the fitness of each individual in the offspring
3. Selection of the next generation of fittest individuals by means of the NSGA-II sorting and selection operator. Briefly, the NSGA-II operator sorts the group of individuals made up of the parent population and the offspring into ranks of non-dominated solutions, i.e. all the non-dominated solutions in the pool are assigned to the first rank; all the non-dominated solutions in the pool minus the solutions in the first rank are assigned to the second rank and so on. Finally, to break the ties within each rank, a measure of crowding is used to measure the density around each solution, preferring those solutions in less crowded areas of each rank; this is to ensure enough exploration of different trade-offs between the single objectives of the fitness function. After sorting, the top $k_{pop}$ individuals in the rank are chosen to form the population of the next generation.


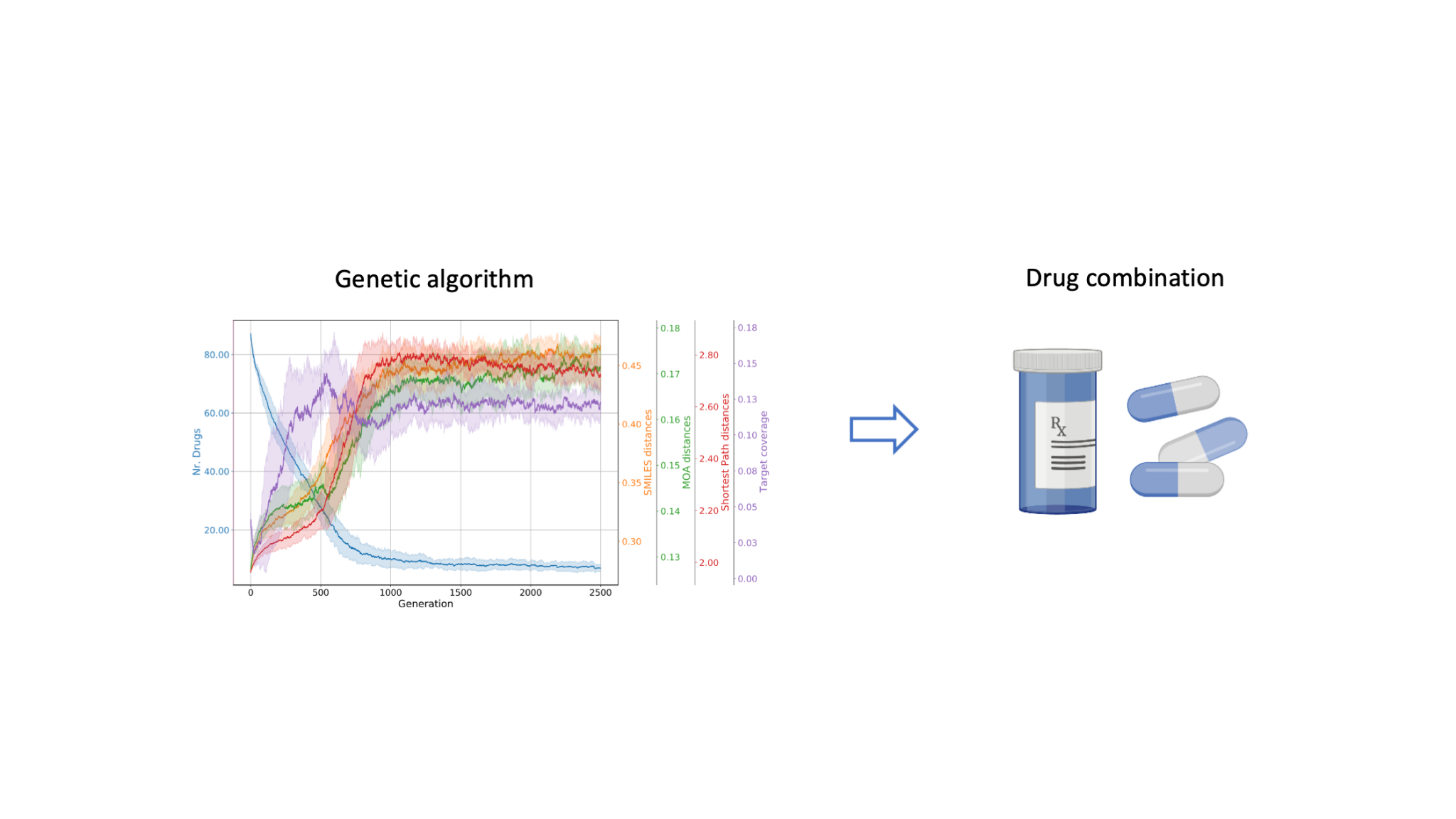


*Figure S3 - In Module 3, the user can exploit the evaluations computed in Module 2 in order to identify drugs candidate to repurposing and combination therapy for the disease under study.*

[Bibliography](https://sciwheel.com/work/bibliography)

[Csardi G, Nepusz T. 2006. The igraph software package for complex network research. *InterJournalComplex Systems,*. 1695](https://sciwheel.com/work/bibliography/13648543)

[De Rainville F-M, Fortin F-A, Gardner M-A, Parizeau M, Gagné C. 2012. DEAP: A python framework for evolutionary algorithms. *Proceedings of the Fourteenth International Conference on Genetic and Evolutionary Computation Conference Companion - GECCO Companion ’12*, p. 85. New York, New York, USA: ACM Press](https://sciwheel.com/work/bibliography/7565056)

[Duncan Temple Lang and the CRAN Team. 2019. *XML: Tools for  Parsing and Generating XML Within R and S-Plus*. R CRAN](https://sciwheel.com/work/bibliography/14498491)

[Duncan Temple Lang and the CRAN team. 2019. *RCurl: General  Network (HTTP/FTP/...) Client Interface for R.* R CRAN](https://sciwheel.com/work/bibliography/14503069)

[Grinberg, M. 2018. *Flask Web Development: Developing Web Applications with Python.* Reilly Media, Inc.](https://sciwheel.com/work/bibliography/14498864)

[H. Pagès, M. Lawrence and P. Aboyoun. 2019. *S4Vectors:  Foundation of Vector-like and List-like Containers in  Bioconductor*. R Bioconductor](https://sciwheel.com/work/bibliography/14498469)

[Hunter JD. 2007. Matplotlib: A 2D Graphics Environment. *Comput. Sci. Eng.* 9(3):90–95](https://sciwheel.com/work/bibliography/1310480)

[Jurman G, Visintainer R, Filosi M, Riccadonna S, Furlanello C. 2015. The HIM glocal metric and kernel for network comparison and classification. *2015 IEEE International Conference on Data Science and Advanced Analytics (DSAA)*, pp. 1–10. IEEE](https://sciwheel.com/work/bibliography/7910061)

[Meyer PE, Lafitte F, Bontempi G. 2008. minet: A R/Bioconductor package for inferring large transcriptional networks using mutual information. *BMC Bioinformatics*. 9:461](https://sciwheel.com/work/bibliography/950850)

[Microsoft and Steve Weston. 2017. *Foreach: Provides Foreach  Looping Construct for R*. R CRAN](https://sciwheel.com/work/bibliography/14498528)

[Microsoft Corporation and Steve Weston. 2018. *DoParallel:  Foreach Parallel Adaptor for the “parallel” Package.* R CRAN](https://sciwheel.com/work/bibliography/14498519)

[Pedregosa et al.,. 2011. Scikit-learn: Machine Learning in Python. *J Mach Learn Res*. 12:2825–30](https://sciwheel.com/work/bibliography/14498897)

[R Core Team. 2019. *R: A Language and Environment for  Statistical Computing.* . R Foundation for Statistical Computing, Vienna, Austria.](https://sciwheel.com/work/bibliography/14503047)

[Tosco P, Stiefl N, Landrum G. 2014. The integration of Open3DTOOLS into the RDKit and KNIME. *J. Cheminform.* 6(S1):](https://sciwheel.com/work/bibliography/14498608)

[Waskom M. 2021. seaborn: statistical data visualization. *JOSS*. 6(60):3021](https://sciwheel.com/work/bibliography/11026692)
