# Supplementary material for: DREAM: an R package for druggability evaluation of human complex diseases
Source: Bioinformatics. 2023 Jul 20;39(7):btad442. doi: 10.1093/bioinformatics/btad442 (PMC10374489; doi:10.1093/bioinformatics/btad442)
Supplement: btad442_Supplementary_Data [file btad442_supplementary_data.zip › Supplementary_file_2.docx]

## DREAM: an R package for druggability evaluation of human complex diseases.

Antonio Federico^1,2,†^, Michele Fratello^1,†^, Alisa Pavel^1^, Lena Möbus^1^, Giusy del Giudice^1^, Angela Serra^1,2^, Dario Greco^1,2,3,4,*^

^1^ Finnish Hub for Development and Validation of Integrated Approaches (FHAIVE), Faculty of Medicine and Health Technology, Tampere University, 33100 Tampere, Finland

^2^ Tampere Institute for Advanced Study

^3^ Division of Pharmaceutical Biosciences, Faculty of Pharmacy, University of Helsinki, 00100 Helsinki, Finland

^4^ Institute of Biotechnology, University of Helsinki, 00100 Helsinki, Finland

^*^ Author to whom correspondence should be addressed.

^†^ These authors contributed equally to this work.

## Results and discussion

Case study

*Experimental setup and data used*

In order to showcase the functionalities of DREAM, we performed a case study on atopic dermatitis (AD) samples deriving from the integration of 12 preprocessed and harmonised public transcriptomics datasets (Table 1). The collected data include samples deriving from both lesional and non-lesional skin of AD patients and are stored in Zenodo (DOI: 10.5281/zenodo.4009497). The aim of the case study is to perform a data-driven druggability evaluation of the AD lesion and eventually derive custom-size sets of drugs that might represent repurposing and drug combination events.

| GEO dataset ID | Citation | Number of AD lesional samples | Number of AD non-lesional samples |
| --- | --- | --- | --- |
| GSE120721 | [(Esaki et al. 2015)](https://sciwheel.com/work/citation?ids=14497870&pre=&suf=&sa=0) | 15 | 15 |
| GSE120899 | [(Simpson et al. 2019)](https://sciwheel.com/work/citation?ids=6322226&pre=&suf=&sa=0) | 18 | 18 |
| GSE27887 | [(Tintle et al. 2011)](https://sciwheel.com/work/citation?ids=14497871&pre=&suf=&sa=0) | 9 | 8 |
| GSE36842 | [(Gittler et al. 2012)](https://sciwheel.com/work/citation?ids=8268650&pre=&suf=&sa=0) | 16 | 8 |
| GSE59294 | [(Hamilton et al. 2014)](https://sciwheel.com/work/citation?ids=4205723&pre=&suf=&sa=0) | 15 | 8 |
| GSE99802 | [(Brunner et al. 2017)](https://sciwheel.com/work/citation?ids=9507625&pre=&suf=&sa=0) | 59 | 53 |
| GSE130588 | [(Guttman-Yassky et al. 2019)](https://sciwheel.com/work/citation?ids=9183592&pre=&suf=&sa=0) | 50 | 41 |
| GSE32924 | [(Suárez-Fariñas et al. 2011)](https://sciwheel.com/work/citation?ids=7844037&pre=&suf=&sa=0) | 13 | 12 |
| GSE58558 | [(Khattri et al. 2014)](https://sciwheel.com/work/citation?ids=5892340&pre=&suf=&sa=0) | 18 | 16 |
| GSE95759 | [(Malik et al. 2017)](https://sciwheel.com/work/citation?ids=14497876&pre=&suf=&sa=0) | 4 | 3 |
| GSE107361 | [(Brunner et al. 2018)](https://sciwheel.com/work/citation?ids=11557903&pre=&suf=&sa=0) | 37 | 66 |
| GSE5667 | [(Plager et al. 2007)](https://sciwheel.com/work/citation?ids=14497878&pre=&suf=&sa=0) | 83 | 294 |

*Table S1 - Publicly available transcriptomics datasets included in the case study.*

*Identification of AD-relevant genes and inference of lesional and non-lesional skin co-expression networks.*

In order to account for the batch effect deriving from the integration of different datasets, all of the collected datasets were combined for c[ross-platform normalisation.](https://www.ncbi.nlm.nih.gov/pmc/articles/PMC4996376/) In particular, the *pamr* R package (version *1.56.1*) [(T. Hastie, R. Tibshirani, Balasubramanian Narasimhan and Gil Chu 2019)](https://sciwheel.com/work/citation?ids=14497796&pre=&suf=&sa=0) was used to mean-adjust the combined microarray data based on a batch variable representing the different datasets downloaded from GEO (<https://www.ncbi.nlm.nih.gov/geo/>).

Therefore, we compared gene expression levels of the lesional samples with the one of the non-lesional ones in order to identify AD-relevant genes. To this aim, we utilised the *get_informative_genes* function, by setting *"wilcoxon + var.test"* as statistical test for the analysis. Through this function we performed 1) a non-parametric hypothesis test (Wilcoxon test) in order to identify substantial differences between the expression levels of the lesional and the non-lesional skin samples and 2) a F-test on the expression variance between the lesional and the non-lesional counterpart. As a result, we obtained that 1,724 genes are to be considered relevant for the disease and, thus, included in subsequent analyses.

The expression values of this set of genes was, then, utilised to infer the co-expression network of the lesional skin. The co-expression network was inferred through the use of INfORM [(Marwah et al. 2018)](https://sciwheel.com/work/citation?ids=4827929&pre=&suf=&sa=0), a tool aimed at the inference of robust co-expression networks through an ensemble approach that we embedded within the DREAM package. The algorithms selected in INfORM to infer the DN network were clr [(Faith et al. 2007)](https://sciwheel.com/work/citation?ids=811307&pre=&suf=&sa=0), ARACNE [(Margolin et al. 2006)](https://sciwheel.com/work/citation?ids=828215&pre=&suf=&sa=0) and MRNET [(Meyer et al. 2007)](https://sciwheel.com/work/citation?ids=2038934&pre=&suf=&sa=0), while the estimation metrics of correlation were Pearson correlation, Spearman correlation, Kendall correlation, empirical mutual information, Miller-Madow asymptotic bias corrected empirical estimator, Schurmann-Grassberger estimate of the entropy of a Dirichlet probability distribution and a shrinkage estimate of the entropy of a Dirichlet probability distribution, as implemented in the R minet package [(Meyer et al. 2008)](https://sciwheel.com/work/citation?ids=950850&pre=&suf=&sa=0). The output of the INfORM algorithm is an undirected binary network.

This network encompasses 1,724 nodes and 190,210 edges.

*Investigated drugs, networks and drug properties*

In the present study, we took advantage of publicly available transcriptome profiles of the LINCS1000 project [(Stathias et al. 2020)](https://sciwheel.com/work/citation?ids=7769529&pre=&suf=&sa=0) (GEO ID: GSE92742).

We performed our investigation on the batch of 21 drugs that have been tested on induced pluripotent stem cells (iPSC) FIBRNPC. The dose and time point of drug perturbation considered in this case study are 10 𝜇m and 24h (Table 2). In order to represent the mechanism of action (MOA) of the drugs on FIBRNPC cells, we inferred co-expression networks by including only the transcriptional signatures of the L1000 landmark genes (for more information, please read [(Stathias et al. 2020)](https://sciwheel.com/work/citation?ids=7769529&pre=&suf=&sa=0)). Such signatures were utilised to infer drug co-expression networks through the use of INfORM as described in the previous paragraph.

| Drug | Pharmaceutical category (ATC code) | Molecular target | Experimental testing for AD treatment | Clinical trials for AD treatment (clinicaltrials.gov and PMIDs) |
| --- | --- | --- | --- | --- |
| Bezafibrate | C10AB02 | PPARG | [(Blunder et al. 2021)](https://sciwheel.com/work/citation?ids=14497821&pre=&suf=&sa=0) | Not available |
| Caffeine | D11AX26, N06BC01 | ADORA1, ADORA2B, GCKR, CYP2B6 | [(Alashqar 2019)](https://sciwheel.com/work/citation?ids=14497844&pre=&suf=&sa=0), [(Kaplan et al. 1978)](https://sciwheel.com/work/citation?ids=14497845&pre=&suf=&sa=0), [(Kaplan et al. 1977)](https://sciwheel.com/work/citation?ids=14497809&pre=&suf=&sa=0), [(Siegel et al. 1978)](https://sciwheel.com/work/citation?ids=14497847&pre=&suf=&sa=0) | NCT02647086, NCT03556592, NCT04562116 |
| Calcifediol | A11CC06, H05BX05 | VDR | [(Berents et al. 2016)](https://sciwheel.com/work/citation?ids=14497851&pre=&suf=&sa=0), [(Weinhold et al. 2016)](https://sciwheel.com/work/citation?ids=14497852&pre=&suf=&sa=0), [(Sánchez-Armendáriz et al. 2018)](https://sciwheel.com/work/citation?ids=6834070&pre=&suf=&sa=0) | NCT04354207, [(Berents et al. 2016)](https://sciwheel.com/work/citation?ids=14497851&pre=&suf=&sa=0), [(Sánchez-Armendáriz et al. 2018)](https://sciwheel.com/work/citation?ids=6834070&pre=&suf=&sa=0) |
| Calcitriol | A11CC04, D05AX03 | VDR | [(Tukaj et al. 2019)](https://sciwheel.com/work/citation?ids=14497854&pre=&suf=&sa=0), [(Bothou et al. 2018)](https://sciwheel.com/work/citation?ids=14497853&pre=&suf=&sa=0), [(Berents et al. 2016)](https://sciwheel.com/work/citation?ids=14497851&pre=&suf=&sa=0), [(Sánchez-Armendáriz et al. 2018)](https://sciwheel.com/work/citation?ids=6834070&pre=&suf=&sa=0) | [(Berents et al. 2016)](https://sciwheel.com/work/citation?ids=14497851&pre=&suf=&sa=0), [(Sánchez-Armendáriz et al. 2018)](https://sciwheel.com/work/citation?ids=6834070&pre=&suf=&sa=0) |
| Isradipine | C08CA03 | CACNA1S | Not available | Not available |
| Metformin | A10BA02 | NDUFAF4 | [(Wang et al. 2020)](https://sciwheel.com/work/citation?ids=14497861&pre=&suf=&sa=0), [(Choi et al. 2020)](https://sciwheel.com/work/citation?ids=14497860&pre=&suf=&sa=0) | Not available |
| Mifepristone | G03XB01 | PGR | Not available | Not available |
| Nimodipine | C08CA06 | CACNA1S | Not available | Not available |
| Pimozide | N05AG02 | DRD1 | [(Lorenzo & Koo 2004)](https://sciwheel.com/work/citation?ids=14497858&pre=&suf=&sa=0), [(Patel et al. 2021)](https://sciwheel.com/work/citation?ids=14497864&pre=&suf=&sa=0) | Not available |
| Pioglitazone | A10BG03 | PPARG | [(Espinoza et al. 2020)](https://sciwheel.com/work/citation?ids=11891927&pre=&suf=&sa=0) | NCT01695707 (withdrawn) |
| Progesterone | G03DA04 | PGR | Not available | Not available |
| Rimonabant | A08AX01 | CNR1 | [(Karsak et al. 2007)](https://sciwheel.com/work/citation?ids=4309924&pre=&suf=&sa=0), [(Ramer & Hinz 2022)](https://sciwheel.com/work/citation?ids=14497863&pre=&suf=&sa=0)  [(Malfitano et al. 2011)](https://sciwheel.com/work/citation?ids=6256609&pre=&suf=&sa=0) | Not available |
| Risperidone | N05AX08 | HTR2C | [(Behshad et al. 2008)](https://sciwheel.com/work/citation?ids=14497859&pre=&suf=&sa=0) | Not available |
| Rosiglitazone | A10BG02 | PPARG | Not available | Not available |
| Sorafenib | L01EX02 | RET | Not available | Not available |
| Suramin | P01CX02 | FGF1 | [(Alyoussef 2015)](https://sciwheel.com/work/citation?ids=14497818&pre=&suf=&sa=0) | Not available |
| Tacedinaline | Not available | HDAC7, HDAC9, HDAC11 | Not available | Not available |
| Tanespimycin | Not available | HSP90AB1 | Not available | Not available |
| Trifluoperazine | N05AB06 | HTR2C | Not available | Not available |
| Troglitazone | A10BG01 | PPARG | [(Dębińska 2021)](https://sciwheel.com/work/citation?ids=14497862&pre=&suf=&sa=0) | Not available |
| Verapamil | C08DA01 | CACNA1S | Not available | Not available |

*Table S2 - Compounds investigated in the case study along with their molecular targets. The table also reports possible experimental evidence of effectiveness of the drug in the therapeutic management of atopic dermatitis and existing clinical trials.*

*Identification of AD-relevant drug combinations.*

The DREAM algorithm predicted 18 drugs to have a potential therapeutic application in atopic dermatitis. As expected, various prioritised compounds have long been used in skin treatment, including caffeine and vitamin D active metabolites [(Kaplan 1977)](https://sciwheel.com/work/citation?ids=14509879&pre=&suf=&sa=0) [(Mesquita et al. 2013)](https://sciwheel.com/work/citation?ids=13455897&pre=&suf=&sa=0), and are currently in clinical trials for atopic dermatitis (Table 2).

More importantly, the results of DREAM were able to capture the two most important axes of the pathogenesis of atopic dermatitis.

First, atopic dermatitis is characterised by strong immunological and inflammatory components [(Brunner et al. 2017)](https://sciwheel.com/work/citation?ids=9507625&pre=&suf=&sa=0). In this light, anti-inflammatory and immunomodulating drugs, such as progesterone, are pivotal targets in managing both patients with early-onset and with chronic disease [(Fedotcheva et al. 2022)](https://sciwheel.com/work/citation?ids=14551775&pre=&suf=&sa=0). DREAM also predicts the repurposing of drugs with narrow therapeutic spectrum to date, like suramin, an antiparasitic with unclear molecular effect. Based on studies trying to clarify its role on parasite infections, we hypothesise that suramin therapeutic potential is mediated by the inhibition of mast cells, thus limiting the elevation of IgE that drives the inflammatory reaction. Indeed, Kawakami et al. suggested that mast cells may be involved in the pathogenesis of atopic dermatitis, by secreting a broad set of pro-inflammatory mediators and sensitization to allergens [(Kawakami et al. 2009)](https://sciwheel.com/work/citation?ids=6551336&pre=&suf=&sa=0).

Next, atopic dermatitis is characterised by major alterations of lipid metabolism [(Emmert et al. 2021)](https://sciwheel.com/work/citation?ids=13447375&pre=&suf=&sa=0) [(Yin et al. 2022)](https://sciwheel.com/work/citation?ids=14509994&pre=&suf=&sa=0), which contributes to alteration of the epidermal barrier composition. The presence of metabolic drugs, including lipid lowering fibrate and antihypertensive drugs may indicate that targeting the metabolism of skin in AD has indeed a pivotal therapeutic role.


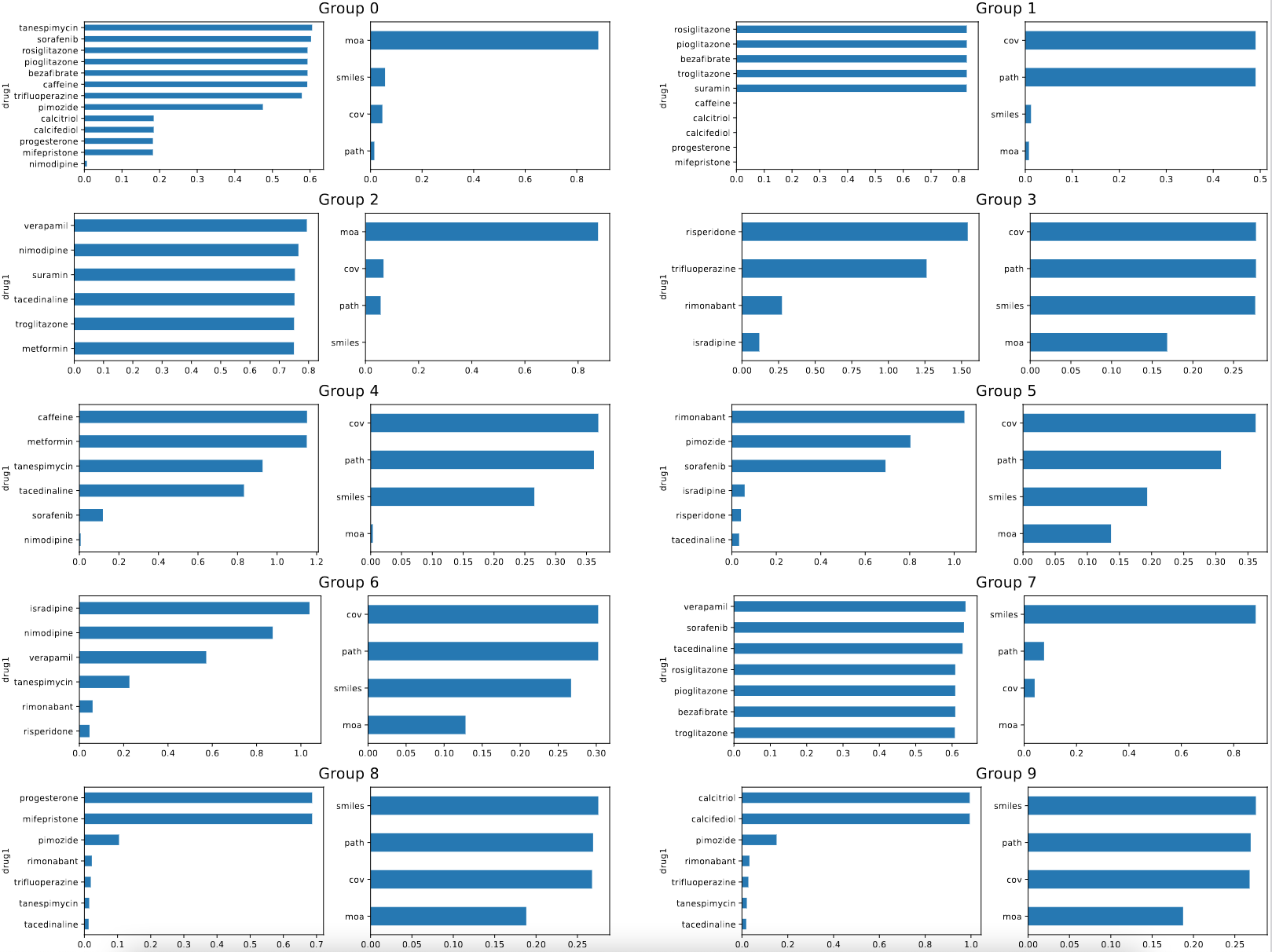


*Figure S4 - Integrative meta-clustering of 21 drugs analysed in this use case, utilising a meta-clustering procedure to integrate information from the multiple layers. Each group is a result of this integration and reflects the drugs with the highest membership. The left column displays the most prominent drugs in each group, while the right column highlights the individual views that contribute the most to each group's formation.*

We further investigated similarity between individual drugs on the different layers, including the mechanistic, chemical and network dimension (<https://github.com/fhaive/dream>). We employed a multi-layered approach to group 21 drugs into clusters based on similarity in their structural, mechanistic, and network coverage features (Figure S4). First, in order to compare and cluster the drugs based on their MOA, we calculated a matrix of similarity among the drugs based on the Hamming-Ipsen-Mikhailov (HIM) distance. Second, to cluster the drugs based on their chemo-structural properties, we extrapolated the drug structures in the form of SMILES (Simplified Molecular Input Line Entry System) and calculated a pairwise Levenstein distance. We, then, evaluated the area of action of the drugs under investigation on the atopic dermatitis network. The area of action for a drug was defined as the union of target genes and their direct interactors on the disease network. Eventually, we computed a matrix reporting the coverage of pairs of drugs. Specifically, the coverage is defined as the non-overlapping area of the DN in terms of direct interactors of drug targets normalised on the total number of nodes composing the DN. Finally, we calculated a matrix of the distances between drug targets of pairs of drugs on the atopic dermatitis network. If one (or both) drug(s) of the pair have more than one target in the atopic dermatitis network, then the average of the distances of all the possible target pairs of the two drugs was computed.

To integrate the results from these layers and identify commonalities among the drug groupings, we utilised an integrative meta-clustering technique. We performed the same process as described in [(Greene & Cunningham 2009)](https://sciwheel.com/work/citation?ids=14547893&pre=&suf=&sa=0), namely we collected all membership matrices (binary matrices with dimensions $d \times c_{l}$, where $d$ is the number of drugs, i.e., 21, and $c_{l}$ is the number of clusters in layer $l$; 1 in position $ij$ means that drug $i$ belongs to cluster $j$ in the $l$-th view) obtained from each layer and assembled into an integrated single matrix by transposing and concatenating them by rows. This new binary matrix $X$ has dimensions $s\times d$, where $s$ is the total number of clusters available across all layers, and each column summarises all the clusters to which each drug belongs across the view. We factorised this matrix by non-negative matrix factorization (NMF) [(Lee & Seung 1999)](https://sciwheel.com/work/citation?ids=387370&pre=&suf=&sa=0) into $k=10$ components. The two output matrices $PH\sim X$ have dimensions $s\times k$ and $k\times d$ respectively. The rows matrix $H^{T}$ can be interpreted as the membership the drugs to each of the components, while matrix $P$ quantifies how much each cluster contributes to the meta-clustering. These findings are summarised in Figure 5, where the left column displays the top drugs in each meta-cluster and the right column indicates the contributing evidence from each layer. Interestingly, Figure 5 highlights that different drugs can belong to different meta-clusters for different reasons, e.g. troglitazone belongs to meta-cluster 7 due to shared chemical structures with other members, while only a subset of those also shared DN network properties as shown in meta-cluster 2.

This information can help to find therapeutic alternatives prioritising the chemical similarity, a shared therapeutic target, the drug mechanism of action or any of their combinations, according to the pharmacological context.

DREAM also predicted drug combinations for atopic dermatitis. Drug combination is an effective therapeutic strategy, as it improves clinical outcomes with lower dosage compared to monotherapy, limiting the possibility of observing adverse effects. Indeed, it has been estimated that around half a million pairwise combinations of FDA approved drugs should be tested for repurposing in almost 3000 diseases [(Cheng et al. 2019)](https://sciwheel.com/work/citation?ids=6964763&pre=&suf=&sa=0).

Therefore, we prioritised combinations composed of 2 drugs, and we discarded combinations including more than four drugs, as their applicability in the clinics may be limited by the compliance of the patient.

Such a threshold has been based on recent literature, which reports four-drug regimen for some infectious disease proving benefits over monotherapy in clinical trials [(Lienhardt et al. 2011)](https://sciwheel.com/work/citation?ids=14497815&pre=&suf=&sa=0). Furthermore, recent studies trying to optimise drug combinations with computational approaches focused on up to four drug combinations [(Larkins-Ford et al. 2022)](https://sciwheel.com/work/citation?ids=13618630&pre=&suf=&sa=0).

We also discarded combinations containing Tacedinaline and Sorafenib, because of their potential toxicity. The former is an antineoplastic drug currently in clinical trials for lung cancer, multiple myeloma and pancreatic cancer. The latter is a kinase inhibitor approved for the treatment of various carcinomas.

After the filtering, DREAM identified 20 combinations composed by 2 drugs, 17 combinations composed by 3 drugs and 4 combinations composed by 4 drugs for atopic dermatitis (Table 3, Figure S5).


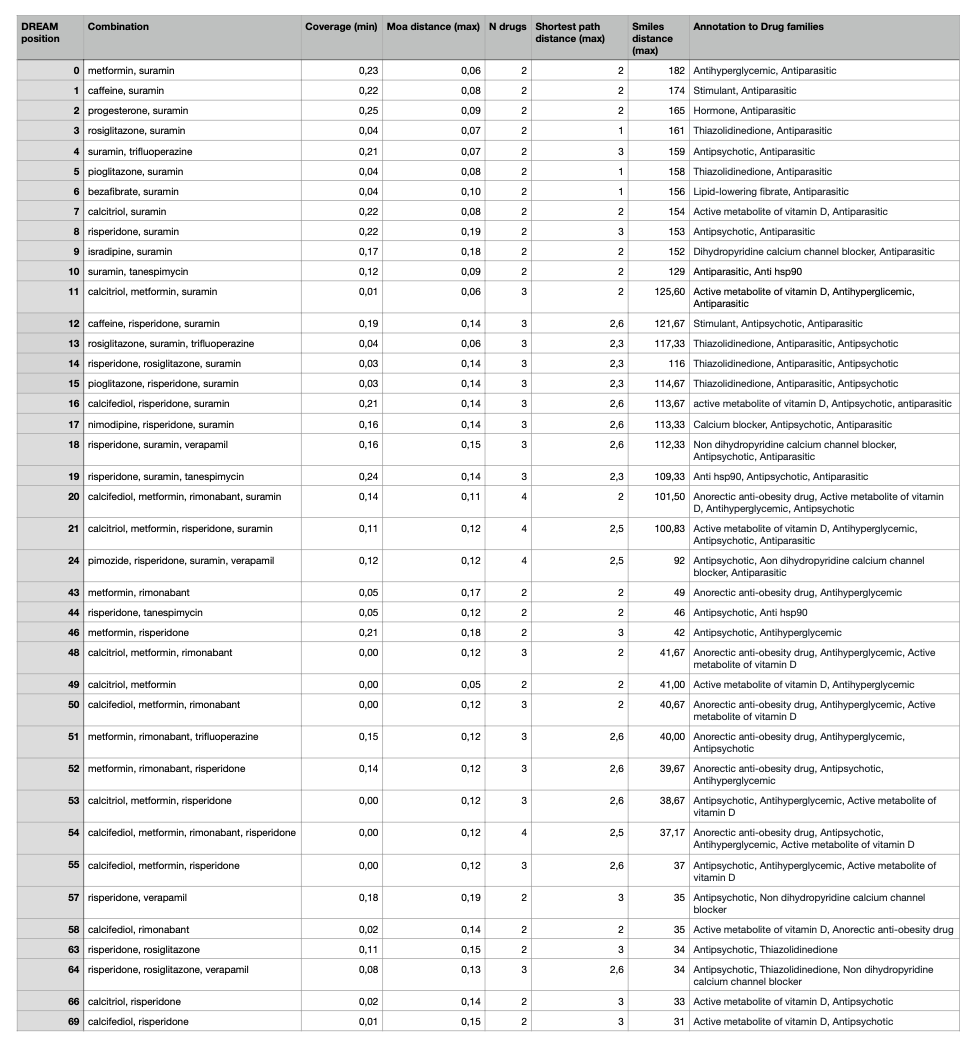


*Table S3. Prioritised drug combinations predicted by DREAM. Network properties and annotations to drug families have been reported.*

Among the selected combinations, metformin and suramin is consistent with our prior discussion on the major components of atopic dermatitis pathogenesis, and further supports the validity of our algorithm. Interestingly, suramin is the most frequently occurring drug over all the combinations. Indeed, it was previously found to alleviate atopic dermatitis symptoms in terms of skin lesion, dermatitis score and scratching behaviour [(Alyoussef 2015)](https://sciwheel.com/work/citation?ids=14497818&pre=&suf=&sa=0). As for metformin, it is the first line of action in patients with type 2 diabetes, especially when obesity is co-occurring. However, metformin is a drug with a very wide range of pharmacological properties and reports of its therapeutic effect on diseases, including inflammation and cancer, are steadily increasing. In particular, its ability to attenuate inflammatory responses in various conditions, by acting on NFkB has already been described [(Chang & Choi 2020)](https://sciwheel.com/work/citation?ids=14257412&pre=&suf=&sa=0). More specifically, it has been proposed that metformin could work as an oral immunosuppressant to chronic inflammatory diseases, including atopic dermatitis.

*
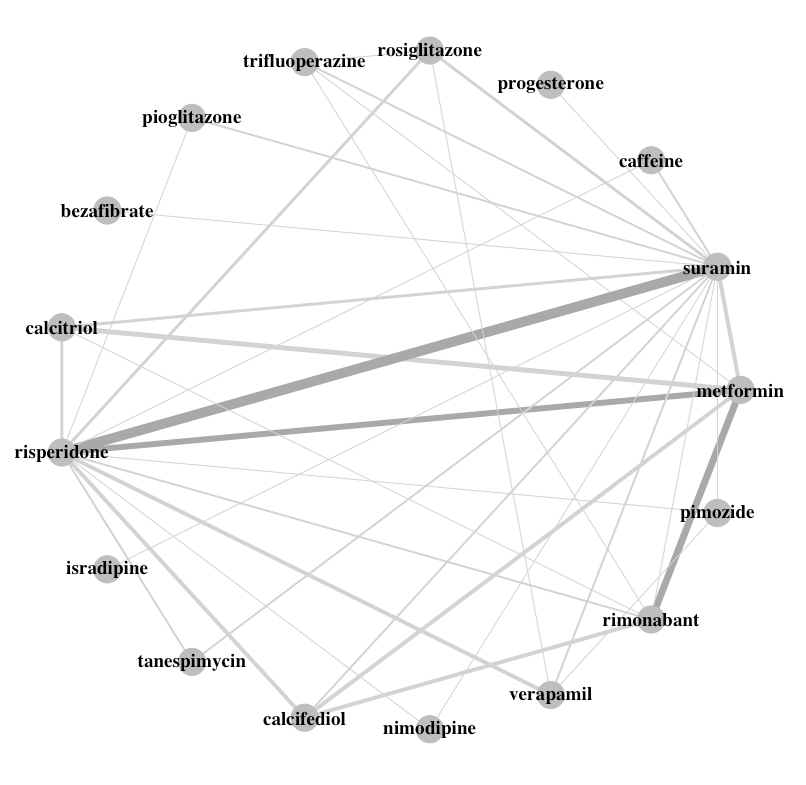
*

*Figure S5 - Graph representation of the most frequent drug combination predicted by DREAM for atopic dermatitis.*

Interestingly, other combinations are present targeting both the metabolism and the inflammatory/immunological compartment.

In detail, the combination of metformin and rimonabant is one of the most frequent produced by the algorithm (Figure S5). Although the role of rimonabant has not been clarified and there are controversial evidences on its role on inflammation and allergy, a study from Malfitano et al. shows that rimonabant reduces keratinocytes viability and has topical anti-inflammatory activity in mice [(Malfitano et al. 2011)](https://sciwheel.com/work/citation?ids=6256609&pre=&suf=&sa=0). This is a very relevant feature, as it is known that keratinocytes proliferate abnormally in the basal and suprabasal layer of lesional skin, but not in the healthy epidermis [(Das et al. 2022)](https://sciwheel.com/work/citation?ids=13306873&pre=&suf=&sa=0).

It is important to acknowledge that diabetes and other metabolic disorders have an elevated incidence in patients with atopic dermatitis [(Olesen et al. 2001)](https://sciwheel.com/work/citation?ids=8658126&pre=&suf=&sa=0). We discuss in the limitation section the problem of the lack of clinical data, which does not allow us to discuss whether the incidence of comorbidities may affect our prediction.

Risperidone and suramin is the combination with the highest MOA and shortest path distance. This is an extremely relevant feature, as it optimises the drug combination both in terms of therapeutic effect and target. Risperidone is an atypical antipsychotic approved for the treatment of schizophrenia, bipolar disorder and depression. It has already been reported that risperidone has a plethora of metabolic and endocrine effects and it was further suggested that low concentration of risperidone can have an impact on the immune system and inflammatory cytokines [(May et al. 2019)](https://sciwheel.com/work/citation?ids=9736183&pre=&suf=&sa=0).

We further exploited the similarity between drugs (Figure S4), and here showcase an example on how it can be utilised to find therapeutic alternatives. Starting from the last combination we discussed, risperidone shares coverage, shortest path similarity, and drug target (Table 2) with trifluoperazine. Although there is no current evidence for the use of trifluoperazine in atopic dermatitis, a recent study from Goda et al. [(Goda et al. 2021)](https://sciwheel.com/work/citation?ids=14555996&pre=&suf=&sa=0) suggested that the compounds may have an effect in suppressing NF-kB and apoptosis. This serves as a proof of concept that the combinations provided by DREAM, together with supporting information and pharmacological knowledge, can drive drug repurposing and selection of relevant mono- and multi-therapy regimes.

*Comparison of DREAM with existing tools*

In the last decade several tools and packages have been developed to leverage drug repurposing and combination predictions to improve the treatment of human complex diseases. However, many of them do not give the possibility to the users to tailor (or improve) the pipelines for different use cases. This drawback results from the lack of code availability, non modifiable pipelines and fixed file formats. It is the case of PREDICT [(Gottlieb et al. 2011)](https://sciwheel.com/work/citation?ids=171558&pre=&suf=&sa=0), which is a method developed with the scope of deriving novel drug indications even in a personalised medicine setting. PREDICT is one of the first and most cited methods attempting to repurpose existing compounds. The predictive system of PREDICT is quite articulated, being based on drug–disease associations, drug–drug and disease–disease similarity metrics, possible drug indications according to their similarity to known drug indications and integration of the similarities to classification features and subsequent classification, the software lacks of a functionality to predict drug combinations. Similarly, DrugRepo [(Wang et al. 2022)](https://sciwheel.com/work/citation?ids=14494441&pre=&suf=&sa=0) is not suitable for drug combination prediction. DrugRepo has been developed as a web application, and as such its functionalities are not exposed and cannot be used in a different manner than provided in its interface and the input formats are restricted. This implies that it is difficult to integrate its functionalities into larger analysis pipelines or to use it in combination with other applications. On the other hand, its simple and intuitive graphical user interface (GUI) greatly improves the suitability for inexperienced researchers. One of the software that implements functions for drug combination prediction is DrugComboRanker [(Huang et al. 2014)](https://sciwheel.com/work/citation?ids=28947&pre=&suf=&sa=0), which is designed to prioritise synergistic drug combinations and uncover their mechanism of action. To the best of our knowledge, DrugComboRanker is the most similar software to DREAM in terms of layers taken into consideration for the scoring of drug combinations (i.e. genomic profiles of the drugs and diseases, and drug similarity based on their intrinsic properties, network-topology evaluations of the combinations). The code of DrugComboRanker is not publicly available and the software is available only upon request.

*Limitations of the study*

This study was conducted on transcriptional profiles annotated in public repositories for which the amount of clinical data made available is limited. The lack of detailed clinical information about the samples hampers the predictive power of the present study, since it does not allow us to infer any meaningful relationship between the obtained druggability profiles and certain clinical parameters of AD patients, including severity of the disease and elapsed time from the first diagnosis, as well as anatomical location of the lesions. Moreover, the lack of information about possible active pharmacological therapy administered to the patients represents a further drawback of the case study. Indeed, the transcriptional signatures underlying the topology of the network model might be influenced by a possible (ongoing or terminated) pharmacological therapy to which some of the patients might be subjected, interfering with the pharmacological footprint that we investigated. Therefore, the lack of an in-depth characterisation of the samples and drug profiles on which the druggability is evaluated, hinders the possibility of translating this study into clinically meaningful predictions. In addition, in order to showcase the functionalities included in the DREAM package, we utilised a set of drugs tested at one time point (24 h) and one dose (10 𝜇m) in induced pluripotent stem cell (FIBRNPC), for which the transcriptional profiles are reported in the L1000 repository. This setting does not allow to infer optimal conditions to carry out experimental validation of the drug predictions obtained in this case study and should be considered as suggestive.

[Bibliography](https://sciwheel.com/work/bibliography)

[Alashqar MB. 2019. Caffeine in the treatment of atopic dermatitis and psoriasis: A review. *J of Skin*. 3(2):59](https://sciwheel.com/work/bibliography/14497844)

[Alyoussef A. 2015. Suramin attenuated inflammation and reversed skin tissue damage in experimentally induced atopic dermatitis in mice. *Inflamm. Allergy Drug Targets*. 13(6):406–10](https://sciwheel.com/work/bibliography/14497818)

[Behshad R, Cooper KD, Korman NJ. 2008. A retrospective case series review of the peroxisome proliferator-activated receptor ligand rosiglitazone in the treatment of atopic dermatitis. *Arch. Dermatol.* 144(1):84–88](https://sciwheel.com/work/bibliography/14497859)

[Berents TL, Lødrup Carlsen KC, Mowinckel P, Sandvik L, Skjerven HO, et al. 2016. Vitamin D levels and atopic eczema in infancy and early childhood in Norway: a cohort study. *Br. J. Dermatol.* 175(1):95–101](https://sciwheel.com/work/bibliography/14497851)

[Blunder S, Pavel P, Minzaghi D, Dubrac S. 2021. Ppardelta in affected atopic dermatitis and psoriasis: A possible role in metabolic reprograming. *Int. J. Mol. Sci.* 22(14):](https://sciwheel.com/work/bibliography/14497821)

[Bothou C, Alexopoulos A, Dermitzaki E, Kleanthous K, Papadimitriou A, et al. 2018. Successful Treatment of Severe Atopic Dermatitis with Calcitriol and Paricalcitol in an 8-Year-Old Girl. *Case Rep. Pediatr.* 2018:9643543](https://sciwheel.com/work/bibliography/14497853)

[Brunner PM, Guttman-Yassky E, Leung DYM. 2017. The immunology of atopic dermatitis and its reversibility with broad-spectrum and targeted therapies. *J. Allergy Clin. Immunol.* 139(4S):S65–76](https://sciwheel.com/work/bibliography/9507625)

[Brunner PM, Israel A, Zhang N, Leonard A, Wen H-C, et al. 2018. Early-onset pediatric atopic dermatitis is characterized by TH2/TH17/TH22-centered inflammation and lipid alterations. *J. Allergy Clin. Immunol.* 141(6):2094–2106](https://sciwheel.com/work/bibliography/11557903)

[Chang J-E, Choi MS. 2020. A molecular perspective on the potential benefits of metformin for the treatment of inflammatory skin disorders. *Int. J. Mol. Sci.* 21(23):](https://sciwheel.com/work/bibliography/14257412)

[Cheng F, Kovács IA, Barabási A-L. 2019. Network-based prediction of drug combinations. *Nat. Commun.* 10(1):1197](https://sciwheel.com/work/bibliography/6964763)

[Choi SY, Lee C, Heo M-J, Choi YM, An I-S, et al. 2020. Metformin ameliorates animal models of dermatitis. *Inflammopharmacology*. 28(5):1293–1300](https://sciwheel.com/work/bibliography/14497860)

[Das P, Mounika P, Yellurkar ML, Prasanna VS, Sarkar S, et al. 2022. Keratinocytes: an enigmatic factor in atopic dermatitis. *Cells*. 11(10):](https://sciwheel.com/work/bibliography/13306873)

[Dębińska A. 2021. New treatments for atopic dermatitis targeting skin barrier repair via the regulation of FLG expression. *J. Clin. Med.* 10(11):](https://sciwheel.com/work/bibliography/14497862)

[Emmert H, Baurecht H, Thielking F, Stölzl D, Rodriguez E, et al. 2021. Stratum corneum lipidomics analysis reveals altered ceramide profile in atopic dermatitis patients across body sites with correlated changes in skin microbiome. *Exp. Dermatol.* 30(10):1398–1408](https://sciwheel.com/work/bibliography/13447375)

[Esaki H, Ewald DA, Ungar B, Rozenblit M, Zheng X, et al. 2015. Identification of novel immune and barrier genes in atopic dermatitis by means of laser capture microdissection. *J. Allergy Clin. Immunol.* 135(1):153–63](https://sciwheel.com/work/bibliography/14497870)

[Espinoza LC, Vera-García R, Silva-Abreu M, Domènech Ò, Badia J, et al. 2020. Topical pioglitazone nanoformulation for the treatment of atopic dermatitis: design, characterization and efficacy in hairless mouse model. *Pharmaceutics*. 12(3):](https://sciwheel.com/work/bibliography/11891927)

[Faith JJ, Hayete B, Thaden JT, Mogno I, Wierzbowski J, et al. 2007. Large-scale mapping and validation of Escherichia coli transcriptional regulation from a compendium of expression profiles. *PLoS Biol.* 5(1):e8](https://sciwheel.com/work/bibliography/811307)

[Fedotcheva TA, Fedotcheva NI, Shimanovsky NL. 2022. Progesterone as an Anti-Inflammatory Drug and Immunomodulator: New Aspects in Hormonal Regulation of the Inflammation. *Biomolecules*. 12(9):](https://sciwheel.com/work/bibliography/14551775)

[Gittler JK, Shemer A, Suárez-Fariñas M, Fuentes-Duculan J, Gulewicz KJ, et al. 2012. Progressive activation of T(H)2/T(H)22 cytokines and selective epidermal proteins characterizes acute and chronic atopic dermatitis. *J. Allergy Clin. Immunol.* 130(6):1344–54](https://sciwheel.com/work/bibliography/8268650)

[Goda AE, Elenany AM, Elsisi AE. 2021. Novel in vivo potential of trifluoperazine to ameliorate doxorubicin-induced cardiotoxicity involves suppression of NF-κB and apoptosis. *Life Sci.* 283:119849](https://sciwheel.com/work/bibliography/14555996)

[Gottlieb A, Stein GY, Ruppin E, Sharan R. 2011. PREDICT: a method for inferring novel drug indications with application to personalized medicine. *Mol. Syst. Biol.* 7:496](https://sciwheel.com/work/bibliography/171558)

[Greene D, Cunningham P. 2009. A matrix factorization approach for integrating multiple data views. In *Machine Learning and Knowledge Discovery in Databases*, Vol. 5781, eds. W Buntine, M Grobelnik, D Mladenić, J Shawe-Taylor, pp. 423–38. Berlin, Heidelberg: Springer Berlin Heidelberg](https://sciwheel.com/work/bibliography/14547893)

[Guttman-Yassky E, Bissonnette R, Ungar B, Suárez-Fariñas M, Ardeleanu M, et al. 2019. Dupilumab progressively improves systemic and cutaneous abnormalities in patients with atopic dermatitis. *J. Allergy Clin. Immunol.* 143(1):155–72](https://sciwheel.com/work/bibliography/9183592)

[Hamilton JD, Suárez-Fariñas M, Dhingra N, Cardinale I, Li X, et al. 2014. Dupilumab improves the molecular signature in skin of patients with moderate-to-severe atopic dermatitis. *J. Allergy Clin. Immunol.* 134(6):1293–1300](https://sciwheel.com/work/bibliography/4205723)

[Huang L, Li F, Sheng J, Xia X, Ma J, et al. 2014. DrugComboRanker: drug combination discovery based on target network analysis. *Bioinformatics*. 30(12):i228-36](https://sciwheel.com/work/bibliography/28947)

[Kaplan RJ, Daman L, Rosenberg EW, Feigenbaum S. 1977. Treatment of atopic dermatitis with topically applied caffeine--a follow-up report. *Arch. Dermatol.* 113(1):107](https://sciwheel.com/work/bibliography/14497809)

[Kaplan RJ, Daman L, Rosenberg EW, Feigenbaum S. 1978. Topical use of caffeine with hydrocortisone in the treatment of atopic dermatitis. *Arch. Dermatol.* 114(1):60–62](https://sciwheel.com/work/bibliography/14497845)

[Kaplan RJ. 1977. Treatment of Atopic Dermatitis With Topically Applied Caffeine— A Follow-up Report. *Arch. Dermatol.* 113(1):107](https://sciwheel.com/work/bibliography/14509879)

[Karsak M, Gaffal E, Date R, Wang-Eckhardt L, Rehnelt J, et al. 2007. Attenuation of allergic contact dermatitis through the endocannabinoid system. *Science*. 316(5830):1494–97](https://sciwheel.com/work/bibliography/4309924)

[Kawakami T, Ando T, Kimura M, Wilson BS, Kawakami Y. 2009. Mast cells in atopic dermatitis. *Curr. Opin. Immunol.* 21(6):666–78](https://sciwheel.com/work/bibliography/6551336)

[Khattri S, Shemer A, Rozenblit M, Dhingra N, Czarnowicki T, et al. 2014. Cyclosporine in patients with atopic dermatitis modulates activated inflammatory pathways and reverses epidermal pathology. *J. Allergy Clin. Immunol.* 133(6):1626–34](https://sciwheel.com/work/bibliography/5892340)

[Larkins-Ford J, Degefu YN, Van N, Sokolov A, Aldridge BB. 2022. Design principles to assemble drug combinations for effective tuberculosis therapy using interpretable pairwise drug response measurements. *Cell Rep. Med.* 3(9):100737](https://sciwheel.com/work/bibliography/13618630)

[Lee DD, Seung HS. 1999. Learning the parts of objects by non-negative matrix factorization. *Nature*. 401(6755):788–91](https://sciwheel.com/work/bibliography/387370)

[Lienhardt C, Cook SV, Burgos M, Yorke-Edwards V, Rigouts L, et al. 2011. Efficacy and safety of a 4-drug fixed-dose combination regimen compared with separate drugs for treatment of pulmonary tuberculosis: the Study C randomized controlled trial. *JAMA*. 305(14):1415–23](https://sciwheel.com/work/bibliography/14497815)

[Lorenzo CR, Koo J. 2004. Pimozide in dermatologic practice: a comprehensive review. *Am. J. Clin. Dermatol.* 5(5):339–49](https://sciwheel.com/work/bibliography/14497858)

[Malfitano AM, Sosa S, Laezza C, De Bortoli M, Tubaro A, Bifulco M. 2011. Rimonabant reduces keratinocyte viability by induction of apoptosis and exerts topical anti-inflammatory activity in mice. *Br. J. Pharmacol.* 162(1):84–93](https://sciwheel.com/work/bibliography/6256609)

[Malik K, Ungar B, Garcet S, Dutt R, Dickstein D, et al. 2017. Dust mite induces multiple polar T cell axes in human skin. *Clin. Exp. Allergy*. 47(12):1648–60](https://sciwheel.com/work/bibliography/14497876)

[Margolin AA, Nemenman I, Basso K, Wiggins C, Stolovitzky G, et al. 2006. ARACNE: an algorithm for the reconstruction of gene regulatory networks in a mammalian cellular context. *BMC Bioinformatics*. 7 Suppl 1(Suppl 1):S7](https://sciwheel.com/work/bibliography/828215)

[Marwah VS, Kinaret PAS, Serra A, Scala G, Lauerma A, et al. 2018. Inform: inference of network response modules. *Bioinformatics*. 34(12):2136–38](https://sciwheel.com/work/bibliography/4827929)

[May M, Beauchemin M, Vary C, Barlow D, Houseknecht KL. 2019. The antipsychotic medication, risperidone, causes global immunosuppression in healthy mice. *PLoS ONE*. 14(6):e0218937](https://sciwheel.com/work/bibliography/9736183)

[Mesquita K de C, Igreja AC de SM, Costa IMC. 2013. Atopic dermatitis and vitamin D: facts and controversies. *An. Bras. Dermatol.* 88(6):945–53](https://sciwheel.com/work/bibliography/13455897)

[Meyer PE, Kontos K, Lafitte F, Bontempi G. 2007. Information-theoretic inference of large transcriptional regulatory networks. *EURASIP J. Bioinform. Syst. Biol.* 79879](https://sciwheel.com/work/bibliography/2038934)

[Meyer PE, Lafitte F, Bontempi G. 2008. minet: A R/Bioconductor package for inferring large transcriptional networks using mutual information. *BMC Bioinformatics*. 9:461](https://sciwheel.com/work/bibliography/950850)

[Olesen AB, Juul S, Birkebaek N, Thestrup-Pedersen K. 2001. Association between atopic dermatitis and insulin-dependent diabetes mellitus: a case-control study. *Lancet*. 357(9270):1749–52](https://sciwheel.com/work/bibliography/8658126)

[Patel P, Patel K, Pandher K, Tareen RS. 2021. The role of psychiatric, analgesic, and antiepileptic medications in chronic pruritus. *Cureus*. 13(8):e17260](https://sciwheel.com/work/bibliography/14497864)

[Plager DA, Leontovich AA, Henke SA, Davis MDP, McEvoy MT, et al. 2007. Early cutaneous gene transcription changes in adult atopic dermatitis and potential clinical implications. *Exp. Dermatol.* 16(1):28–36](https://sciwheel.com/work/bibliography/14497878)

[Ramer R, Hinz B. 2022. Cannabinoid Compounds as a Pharmacotherapeutic Option for the Treatment of Non-Cancer Skin Diseases. *Cells*. 11(24):](https://sciwheel.com/work/bibliography/14497863)

[Sánchez-Armendáriz K, García-Gil A, Romero CA, Contreras-Ruiz J, Karam-Orante M, et al. 2018. Oral vitamin D3 5000 IU/day as an adjuvant in the treatment of atopic dermatitis: a randomized control trial. *Int. J. Dermatol.* 57(12):1516–20](https://sciwheel.com/work/bibliography/6834070)

[Siegel FP, Ecanow B, Blake MI. 1978. Caffeine as an adjunct in the treatment of atopic dermatitis. *Arch. Dermatol.* 114(11):1717](https://sciwheel.com/work/bibliography/14497847)

[Simpson EL, Imafuku S, Poulin Y, Ungar B, Zhou L, et al. 2019. A Phase 2 Randomized Trial of Apremilast in Patients with Atopic Dermatitis. *J. Invest. Dermatol.* 139(5):1063–72](https://sciwheel.com/work/bibliography/6322226)

[Stathias V, Turner J, Koleti A, Vidovic D, Cooper D, et al. 2020. LINCS Data Portal 2.0: next generation access point for perturbation-response signatures. *Nucleic Acids Res.* 48(D1):D431–39](https://sciwheel.com/work/bibliography/7769529)

[Suárez-Fariñas M, Tintle SJ, Shemer A, Chiricozzi A, Nograles K, et al. 2011. Nonlesional atopic dermatitis skin is characterized by broad terminal differentiation defects and variable immune abnormalities. *J. Allergy Clin. Immunol.* 127(4):954-64.e1](https://sciwheel.com/work/bibliography/7844037)

[T. Hastie, R. Tibshirani, Balasubramanian Narasimhan and Gil Chu. 2019. *Pam: Prediction Analysis  for Microarrays. R Package Version 1.56.1. Https://CRAN.R-Project.Org/Package=pamr*. CRAN R-project](https://sciwheel.com/work/bibliography/14497796)

[Tintle S, Shemer A, Suárez-Fariñas M, Fujita H, Gilleaudeau P, et al. 2011. Reversal of atopic dermatitis with narrow-band UVB phototherapy and biomarkers for therapeutic response. *J. Allergy Clin. Immunol.* 128(3):583-93.e1](https://sciwheel.com/work/bibliography/14497871)

[Tukaj S, Zillikens D, Kasperkiewicz M. 2019. Topically applied low-dose calcitriol ameliorates atopic eyelid dermatitis. *JAAD Case Reports*. 5(1):5–6](https://sciwheel.com/work/bibliography/14497854)

[Wang M, Qu S, Ma J, Wang X, Yang Y. 2020. Metformin Suppresses LPS-Induced Inflammatory Responses in Macrophage and Ameliorates Allergic Contact Dermatitis in Mice via Autophagy. *Biol. Pharm. Bull.* 43(1):129–37](https://sciwheel.com/work/bibliography/14497861)

[Wang Y, Aldahdooh J, Hu Y, Yang H, Vähä-Koskela M, et al. 2022. DrugRepo: a novel approach to repurposing drugs based on chemical and genomic features. *Sci. Rep.* 12(1):21116](https://sciwheel.com/work/bibliography/14494441)

[Weinhold A, Obeid R, Vogt T, Reichrath J. 2016. Prospective Investigation of 25(OH)D3 Serum Concentration Following UVB Narrow Band Phototherapy in Patients with Psoriasis and Atopic Dermatitis. *Anticancer Res.* 36(3):1439–44](https://sciwheel.com/work/bibliography/14497852)

[Yin H, Qiu Z, Zhu R, Wang S, Gu C, et al. 2022. Dysregulated lipidome of sebum in patients with atopic dermatitis. *Allergy*](https://sciwheel.com/work/bibliography/14509994)
