# Supplementary figures and images for: DREAM: an R package for druggability evaluation of human complex diseases
Source: Bioinformatics. 2023 Jul 20;39(7):btad442. doi: 10.1093/bioinformatics/btad442 (PMC10374489; doi:10.1093/bioinformatics/btad442)

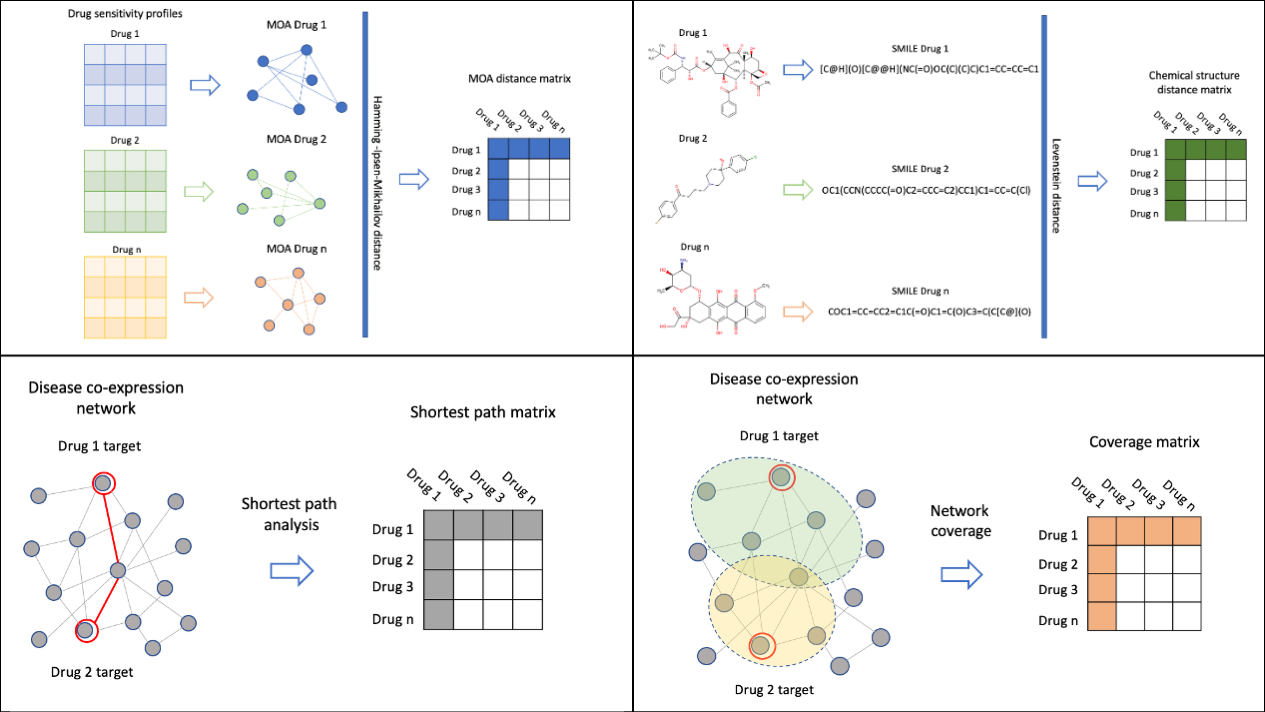

Supplement: btad442_Supplementary_Data [file btad442_supplementary_data.zip › DREAM_Fig_S2.png]

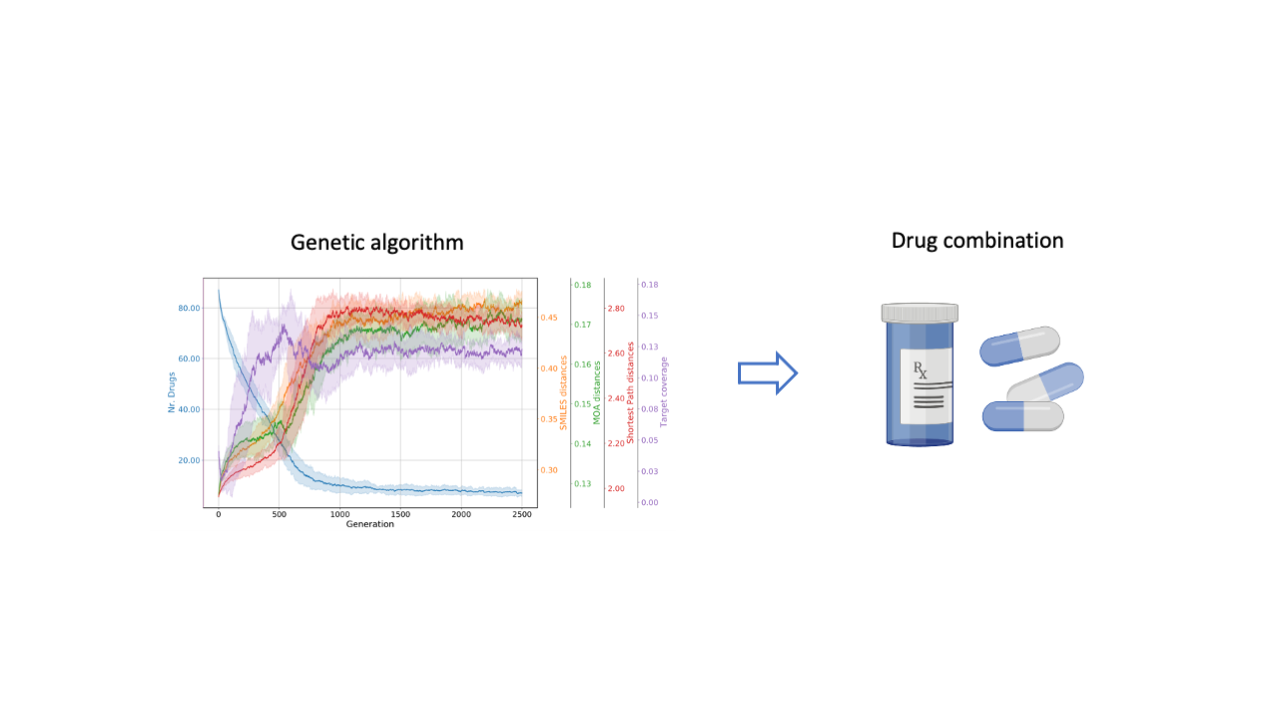

Supplement: btad442_Supplementary_Data [file btad442_supplementary_data.zip › DREAM_Fig_S3.png]

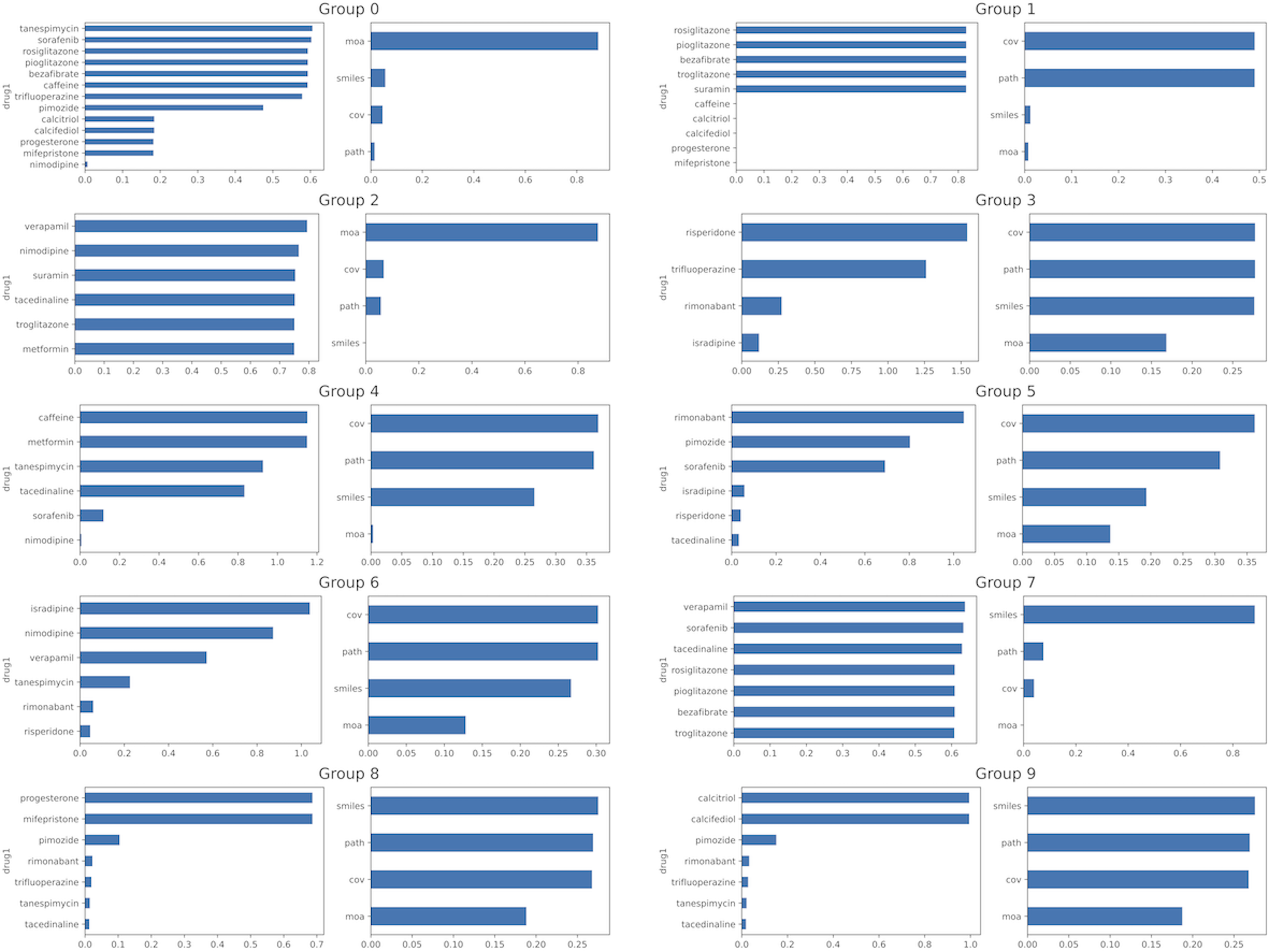

Supplement: btad442_Supplementary_Data [file btad442_supplementary_data.zip › DREAM_Fig_S4.png]

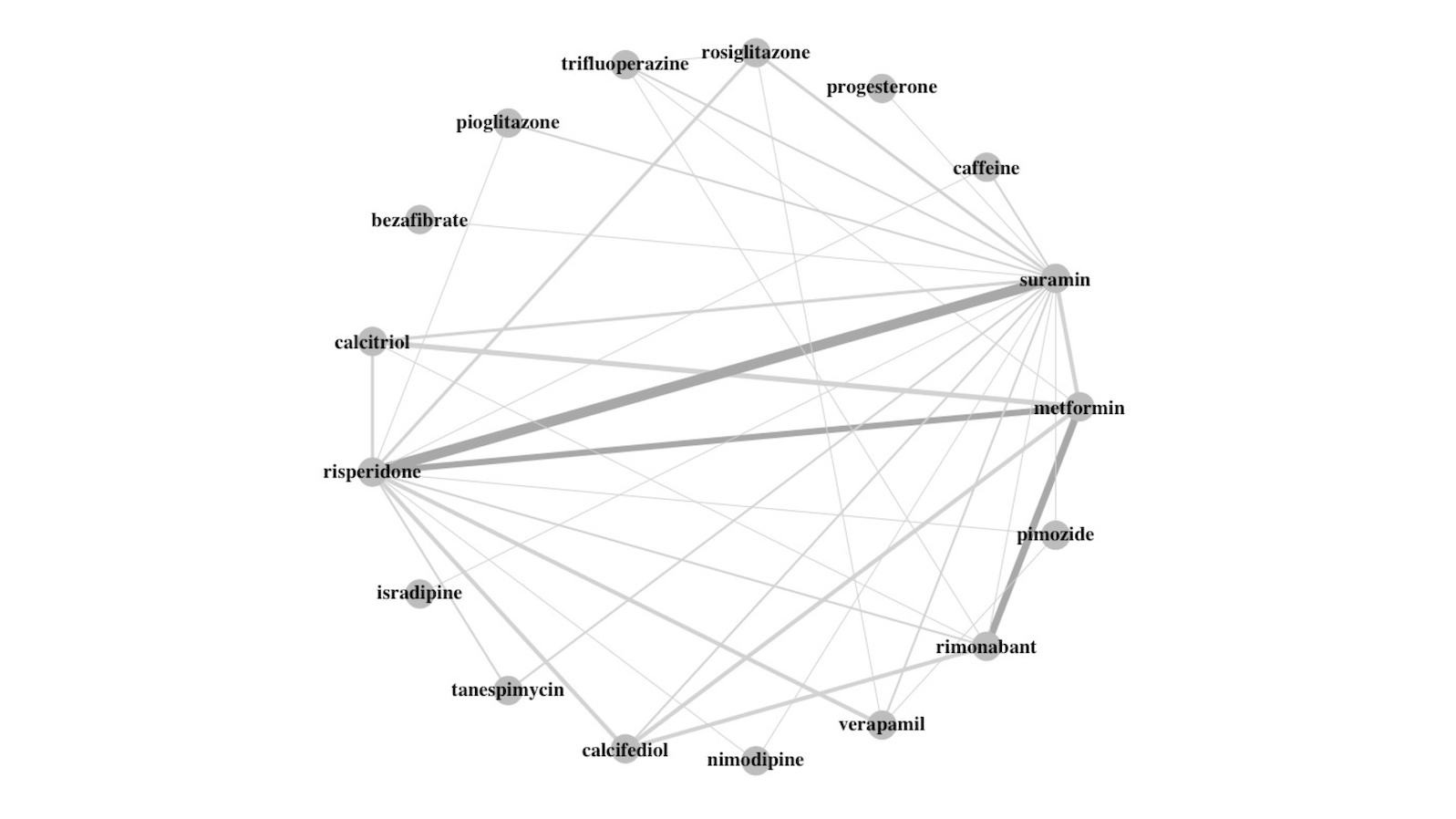

Supplement: btad442_Supplementary_Data [file btad442_supplementary_data.zip › DREAM_Fig_S5.png]

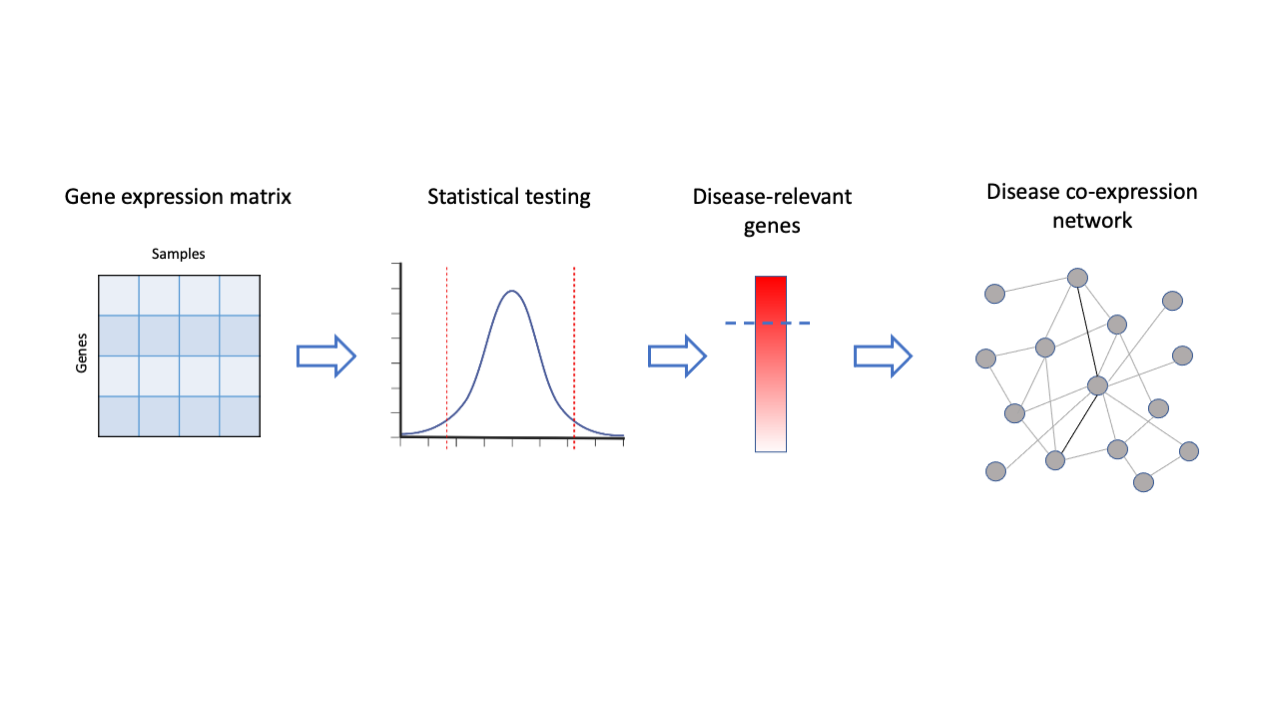

Supplement: btad442_Supplementary_Data [file btad442_supplementary_data.zip › DREAM_Fig_S1.png]
